# Nasopharyngeal Microbiota in South African Infants With Lower Respiratory Tract Infection: A Nested Case-Control Study of the Drakenstein Child Health Study

**DOI:** 10.1093/cid/ciaf184

**Published:** 2025-04-17

**Authors:** Shantelle Claassen-Weitz, Yao Xia, Luke Hannan, Sugnet Gardner-Lubbe, Kilaza S Mwaikono, Stephanie Harris Mounaud, William C Nierman, Lesley Workman, Felix S Dube, Samantha Africa, Fadheela Patel, Veronica Allen, Lemese Ah Tow Edries, Heather J Zar, Mark P Nicol

**Affiliations:** Division of Medical Microbiology, Department of Pathology, Faculty of Health Sciences, University of Cape Town, Cape Town, South Africa; Marshall Centre, Division of Infection and Immunity, School of Biomedical Sciences, University of Western Australia, Perth, Australia; Division of Epidemiology and Biostatistics, School of Public Health and Family Medicine, University of Cape Town, Stellenbosch, South Africa; Department of Statistics and Actuarial Science, Faculty of Economic and Management Sciences, Stellenbosch University, Stellenbosch, South Africa; Computational Biology Group and H3ABioNet, Department of Integrative Biomedical Sciences, University of Cape Town, Cape Town, South Africa; Department of Science and Laboratory Technology, Dar es Salaam Institute of Technology, Dar es Salaam, Tanzania; J. Craig Venter Institute, Rockville, Maryland, USA; J. Craig Venter Institute, Rockville, Maryland, USA; South African Medical Research Council Unit on Child and Adolescent Health, University of Cape Town, Cape Town, South Africa; Institute of Infectious Disease and Molecular Medicine, Faculty of Health Sciences, University of Cape Town, Cape Town, South Africa; Institute of Infectious Disease and Molecular Medicine, Faculty of Health Sciences, University of Cape Town, Cape Town, South Africa; Department of Molecular and Cell Biology, Faculty of Sciences, University of Cape Town, Cape Town, South Africa; Division of Medical Microbiology, Department of Pathology, Faculty of Health Sciences, University of Cape Town, Cape Town, South Africa; Division of Medical Microbiology, Department of Pathology, Faculty of Health Sciences, University of Cape Town, Cape Town, South Africa; Division of Medical Microbiology, Department of Pathology, Faculty of Health Sciences, University of Cape Town, Cape Town, South Africa; Division of Medical Microbiology, Department of Pathology, Faculty of Health Sciences, University of Cape Town, Cape Town, South Africa; South African Medical Research Council Unit on Child and Adolescent Health, University of Cape Town, Cape Town, South Africa; Institute of Infectious Disease and Molecular Medicine, Faculty of Health Sciences, University of Cape Town, Cape Town, South Africa; Department of Paediatrics and Child Health, Red Cross War Memorial Children's Hospital, Cape Town, South Africa; Division of Medical Microbiology, Department of Pathology, Faculty of Health Sciences, University of Cape Town, Cape Town, South Africa; Marshall Centre, Division of Infection and Immunity, School of Biomedical Sciences, University of Western Australia, Perth, Australia

**Keywords:** 16S rRNA gene, infant, lower respiratory tract infection, microbiota, viral

## Abstract

**Background:**

Lower respiratory tract infections (LRTIs) in infants are caused by viral and bacterial infections. We investigated associations between LRTI and nasopharyngeal (NP) viruses and bacteria in South African infants.

**Methods:**

In a birth cohort, LRTI cases were identified prospectively and age-matched with controls. NP swabs were tested using polymerase chain reaction and 16S RNA gene sequencing. We calculated adjusted conditional odds ratios (aORs) and used mixed-effects models to identify differentially abundant taxa and explore viral–bacterial interactions.

**Results:**

A total of 888 case-control samples were tested. Respiratory syncytial virus (RSV) (aOR, 5.69 [95% confidence interval, 3.03–10.69]), human rhinovirus (HRV) (1.47 [1.03–2.09]), parainfluenza virus (3.46 [1.64–7.26]), adenovirus (1.99 [1.08–3.68]), enterovirus (2.32 [1.20–4.46]), *Haemophilus influenzae* (1.72 [1.25–2.37]), *Klebsiella pneumoniae* (2.66 [1.59–4.46]), and high-density *Streptococcus pneumoniae* (1.53 [1.01–2.32]) were associated with LRTI. LRTI was associated with decreased relative abundance of *Dolosigranulum* (q = .001), *Corynebacterium* (q = .091), and *Neisseria* (q = .004). In samples positive for RSV, *Staphylococcus* and *Alloprevotella* relative abundance was higher in controls compared to cases. In samples positive for parainfluenza virus or HRV, *Haemophilus* relative abundance was higher in cases. Detection of cytomegalovirus (CMV) in controls was associated with reduced *Corynebacterium*, *Dolosigranulum*, and *Staphylococcus.*

**Conclusions:**

The associations between bacteria, viruses and LRTIs are similar to those from high-income countries. *Haemophilus* is a major bacterial driver of LRTIs, acting synergistically with viruses. *Dolosigranulum* and *Corynebacterium* may reduce LRTI risk, while *Staphylococcus* may reduce the risk of RSV-related LRTIs. CMV is associated with dysbiotic nasopharyngeal microbiota.

Lower respiratory tract infections (LRTIs) are the leading cause of child death outside of the neonatal period [1], with the burden of infection disproportionately affecting low- and middle-income countries (LMICs) [2].

Infection of the nasopharynx with pathogenic viruses and bacteria frequently precedes LRTI, with local replication and subsequent translocation to the lower respiratory tract [3]. Dysbiotic (imbalanced) nasopharyngeal (NP) bacterial community states may have reduced capability to resist microbial overgrowth and invasion [4]. For example, LRTI caused by respiratory syncytial virus (RSV) has been associated with increased abundance of *Haemophilus influenzae* and *Streptococcus* species and decreased abundance of *Staphylococcus aureus* [5]. *Haemophilus influenzae* and *Streptococcus*-dominated profiles have also been associated with an exaggerated inflammatory response and more severe RSV disease [5].

Risk factors for LRTI, such as malnutrition, maternal human immunodeficiency virus (HIV) infection, lack of exclusive breastfeeding, and indoor air pollution, are more prevalent in LMICs compared to high-income countries [6], which may influence etiology and pathogen interactions. However, studies investigating the association between infant NP bacterial community profiles and LRTI have primarily been performed in high-income settings, and few have measured the bacterial microbiota in parallel with viral testing. We therefore aimed to comprehensively investigate associations between NP bacterial community profiles, viral infection, and LRTI within the first year of life in low-income households in South Africa.

## MATERIALS AND METHODS

### Study Design, Participants, and Specimen Matching

We conducted a case-control study of infants enrolled in the Drakenstein Child Health Study (DCHS), a birth cohort study in a periurban area in South Africa [7]. Ethical approval was received from the Human Research Ethics Committee of the University of Cape Town, South Africa (401/2009 and 585/2015).

All births and hospital care occurred at Paarl Hospital [7]. Mother–infant pairs were followed from birth, with study visits at 6, 10, and 14 weeks and 6, 9, and 12 months. Participants who gave additional consent participated in fortnightly NP sampling across the first year of life. Demographic and clinical data were recorded antenatally (20–28 weeks’ gestation), at birth, and postnatally during scheduled study visits (Supplementary Appendix p. 2). LRTI episodes during the first year of life were identified by active surveillance at local clinics and Paarl Hospital [8] using World Health Organization criteria [9], of cough or difficulty breathing, accompanied by a fast respiratory rate (≥50 breaths per minute, for children 2–11 months old). Mothers were counseled about key respiratory symptoms and advised to contact study staff whenever their infant developed cough or difficulty breathing [10].

NP flocked swabs (FLOQSwab, Copan Diagnostics, Murrieta, California, USA) were collected fortnightly from infants at all timepoints and LRTI events (0–365 + 14 days) (Supplementary Figure 1). Case-control sets consisted of NP specimens from LRTI cases and non-LRTI controls matched 1:1 by birth date (maximum age difference: ±14 days) and study site. Additional information regarding the categorization of case and control specimens is provided in the Supplementary Appendix (pp. 3–4; Supplementary Figure 1).

### Laboratory Procedures

Nasopharyngeal specimens were placed in PrimeStore Molecular Transport medium (Longhorn Vaccines and Diagnostics, Bethesda, Maryland, USA), transported on ice, and stored at −80°C. Nucleic acid extracts (Supplementary Appendix p. 4) were screened for viral and bacterial species using quantitative real-time polymerase chain reaction (qPCR) (Fast-Track Diagnostics Respiratory Pathogens 33 [FTDResp33] assay, Esch-sur-Alzet, Luxembourg) [11]. We applied an organism-specific threshold (>6.9 log_10_ copies/mL) for defining high-density *Streptococcus pneumoniae* that best differentiates cases from controls [12].

We performed short-read 16S ribosomal RNA (rRNA) gene amplicon sequencing on NP specimens where sufficient sample was available. Sequencing runs included comprehensive sequencing controls (Supplementary Appendix pp. 4–5; Supplementary Figure 2) [13]. We measured total 16S rRNA gene copy numbers (16S rRNA gene copies/μL) from nucleic acid extracts using qPCR [13]. Two-step PCR was used to amplify the V4 hypervariable region of the 16S rRNA gene. Pooled libraries were sequenced on Illumina MiSeq using V3 chemistry with 2 × 251 cycles (MiSeq Reagent Kit v3 [600-cycle], Illumina, San Diego, California, USA), (Supplementary Appendix pp. 5–7). We used the DADA2 pipeline (wrapped in the Nextflow algorithm) to filter and trim reads and infer amplicon sequence variants (ASVs) (Supplementary Appendix p. 7). Taxonomy was assigned using the Ribosomal Database Project classifier implementation for DADA2 and SILVA version 138. We used R software version 4.1.2 [14] and RStudio version 2021.09.2 to remove ASVs classified as Eukaryota and ASVs unclassified at kingdom level. We applied a stepwise in silico quality control approach to remove low-quality amplicon data [13] and potential contaminant ASVs (Supplementary Appendix p. 8).

### Statistical Analysis

We calculated conditional odds ratios (ORs, adjusted for age and antibiotic use) and population attributable fractions (PAFs) using the Miettinen formula for each viral and bacterial qPCR target, stratified by age (0–3, >3 to 6, and >6 to 12 months).

Associations between LRTI and NP bacterial load or α-diversity indices (Shannon and Chao1) were assessed using linear regression. Permutational analysis of variance (PERMANOVA) (Bray-Curtis dissimilarity assessed using adonis2 function from *vegan* package) was used to compare overall microbial composition between cases and controls [15]. Regression and PERMANOVA variables included case status, age, and antibiotic exposure.

K-means clustering was done using the 25 most abundant ASVs. We then used logistic regression to model associations between cluster membership and case status, including cluster membership along with absolute abundances of RSV (A/B), parainfluenza virus (PIV), enterovirus, adenovirus, human rhinovirus (HRV), cytomegalovirus (CMV), age, and bacterial load.

We used Microbiome Multivariable Associations with Linear Model (MaAsLin2) version 1.8.0 [16] to identify ASVs that were differentially abundant between cases and controls. Case-control group and age were included as fixed effects. Total sum scaled normalization and log transformation were applied. The results were visualized using Seaborn v0.12 [17] in Python. Subsequently, we chose 4 subsets comprising individuals infected with RSV, HRV, PIV, or CMV (selected due to high prevalence or strong association with LRTI), in MaAsLin2 models with case-control group and age as fixed effects, to identify bacterial taxa associated with LRTI within each subset. We further used MaAsLin2 models with age as a fixed effect to identify ASVs that were associated with detection of the same 4 viral targets, stratified by case or control status.

## RESULTS

### Demographics and Clinical Characteristics

**Figure 1.**
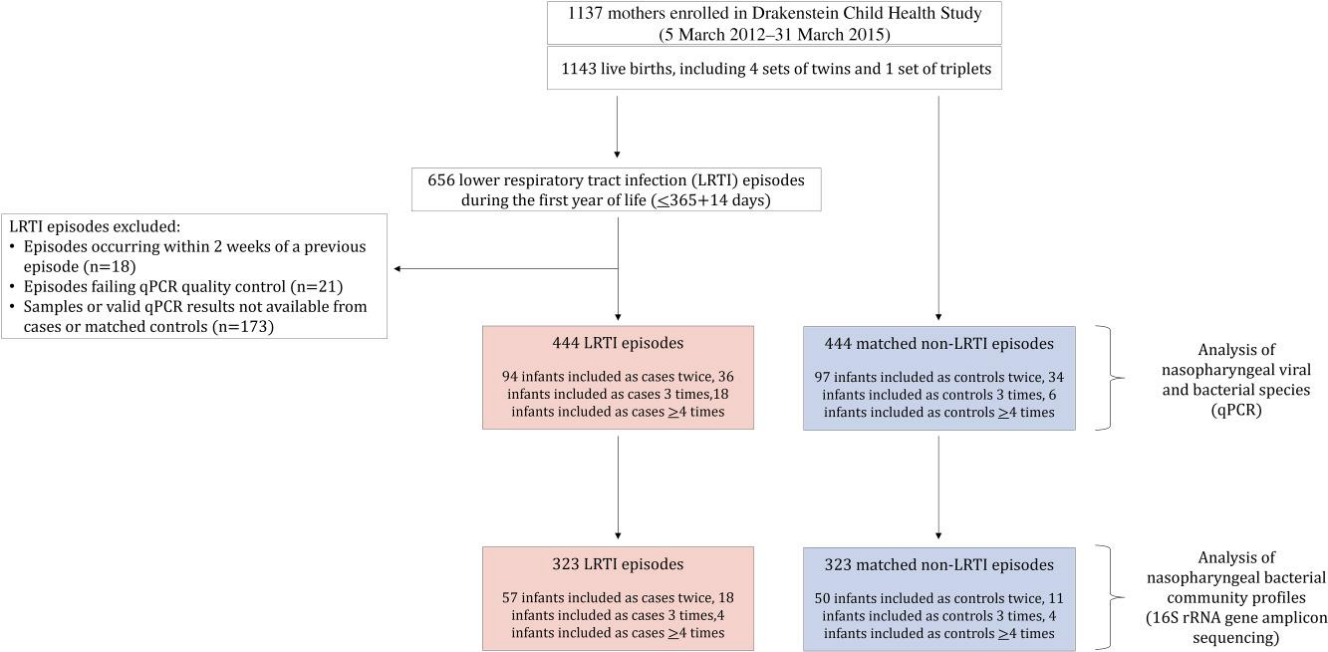
Study flow diagram. Abbreviations: LRTI, lower respiratory tract infection; qPCR, quantitative real-time polymerase chain reaction; rRNA, ribosomal RNA.

Over a 3-year period, 1137 mothers were enrolled with 1143 live births. Cohort retention was high (88.8% [1015/1143] infants at 1 year) [18]. During the first year of life, 656 LRTI episodes were identified (Figure 1). Results from multiplex qPCR were available for 444 case specimens and 444 matched control specimens (from 544 infants) (Figure 1). A subset of these specimens (323 case specimens and 323 control specimens) had high-quality 16S rRNA gene amplicon sequence data available (Supplementary Appendix pp. 13–17).

The median age of NP specimen collection was 139 days (interquartile range [IQR], 81–220 days) (Table 1, Supplementary Table 1). Maternal smoking was identified in 27% (151/544) of participants. Poor socioeconomic status was reflected by low maternal educational attainment (67% of mothers had primary level education only), any parent employment rate (49.6%), and monthly income (<5000 South African rand/283 US dollars per month for 88% of households). Median household size was 4 people (IQR, 3–6). Most infants were delivered by vaginal delivery (80% [442/542]) with 17% (93/544) born premature (<37 weeks’ gestation). Twenty-seven percent (148/544) of infants were HIV exposed but none were HIV infected, and duration of exclusive breastfeeding was short (median duration, 1.4 months [IQR, 0.5–3.0 months]). Immunization coverage for all Expanded Programme on Immunization vaccines was high (>98%).

**Table 1.** Characteristics of Study Population Included in the Lower Respiratory Tract Infection Case-Control Analysis

Characteristic	Overall	Controls	Single Episode of LRTI	≥2 Episodes of LRTI	*P* Value
	N = 544 Children	n = 250 (45.1)	n = 172 (31.1)	n = 132 (23.8)	
Maternal smoking (self-report)					
Yes	151 (27.3)	66 (26.4)	44 (25.6)	41 (31.1)	.522
Maternal education					
Primary level	52 (9.4)	22 (8.8)	15 (8.7)	15 (11.4)	.752
Started secondary level	319 (57.6)	139 (55.6)	99 (57.6)	81 (61.4)	
Completed secondary level	151 (27.3)	73 (29.2)	47 (27.3)	31 (23.5)	
Any tertiary level	32 (5.8)	16 (6.4)	11 (6.4)	5 (3.8)	
Parent employed					
Yes	275 (49.6)	128 (51.2)	86 (50.0)	61 (46.2)	.646
Household income (per month)					
<1000 ZAR	187 (33.8)	78 (31.2)	56 (32.6)	53 (40.2)	.217
1000–5000 ZAR	302 (54.5)	147 (58.8)	91 (52.9)	64 (48.5)	
>5000 ZAR	65 (11.7)	25 (10.0)	25 (14.5)	15 (11.4)	
Household density, median (IQR)	4 (3–6)	4 (3–6)	4 (3–6)	4 (3–6)	.594
Mode of delivery					
Vaginal delivery	442 (79.8)	202 (80.8)	140 (81.4)	100 (75.8)	.594
Season of birth					
Summer	152 (27.4)	77 (30.8)	47 (27.3)	28 (21.2)	.210
Autum	152 (27.4)	62 (24.8)	44 (25.6)	46 (34.9)	
Winter	134 (24.2)	60 (24.0)	47 (27.3)	27 (20.5)	
Spring	116 (20.9)	51 (20.4)	34 (19.8)	31 (23.5)	
WAZ at birth, median (IQR)	−0.3 (−0.0 to 0.4)	−0.2 (−1.0 to 0.4)	−0.4 (−1.0 to 0.3)	−0.3 (−0.9 to 0.4)	.276
Gestational age					
Premature (<37 weeks’ gestation)	93 (16.8)	30 (12.0)	25 (14.5)	38 (28.8)	**<.001**
Sex					
Male	294 (53.1)	118 (47.2)	95 (55.2)	81 (61.4)	.**024**
HIV exposure					.**007**
HIV-exposed, uninfected	148 (26.7)	56 (22.4)	43 (25.0)	49 (37.1)	
Exclusive breastfeeding (months), median (IQR)	1.4 (0.5–3.0)	1.4 (0.7–3.2)	1.0 (0.5–2.8)	1.1 (0.0–3.0)	.302

Data are presented as No. (%) unless otherwise indicated. Values in bold show significant differences (*P* < .05) between cases (single episode of LRTI and ≥2 episodes of LRTI) and controls.

Abbreviations: HIV, human immunodeficiency virus; IQR, interquartile range; LRTI, lower respiratory infection; WAZ, weight-for-age z-score; ZAR, South African rand.

Children who experienced >1 LRTI episode were more likely than children who experienced only a single episode to be born preterm (29% vs 15%, *P* < .001) and to be HIV exposed (37% vs 25%, *P* = .007). Nineteen percent (85/444) of specimens collected at LRTI were from children who had received antibiotics prior to specimen collection; none of the controls received antibiotics (*P* < .001). Among LRTI specimens, 4% were collected in the 14 days prior to LRTI diagnosis; viral detection did not differ between specimens collected at, compared with before, diagnosis (Supplementary Table 2), nor did symptom prevalence (Supplementary Table 3).

### Nasopharyngeal Pathogens Associated With LRTI

**Figure 2.**
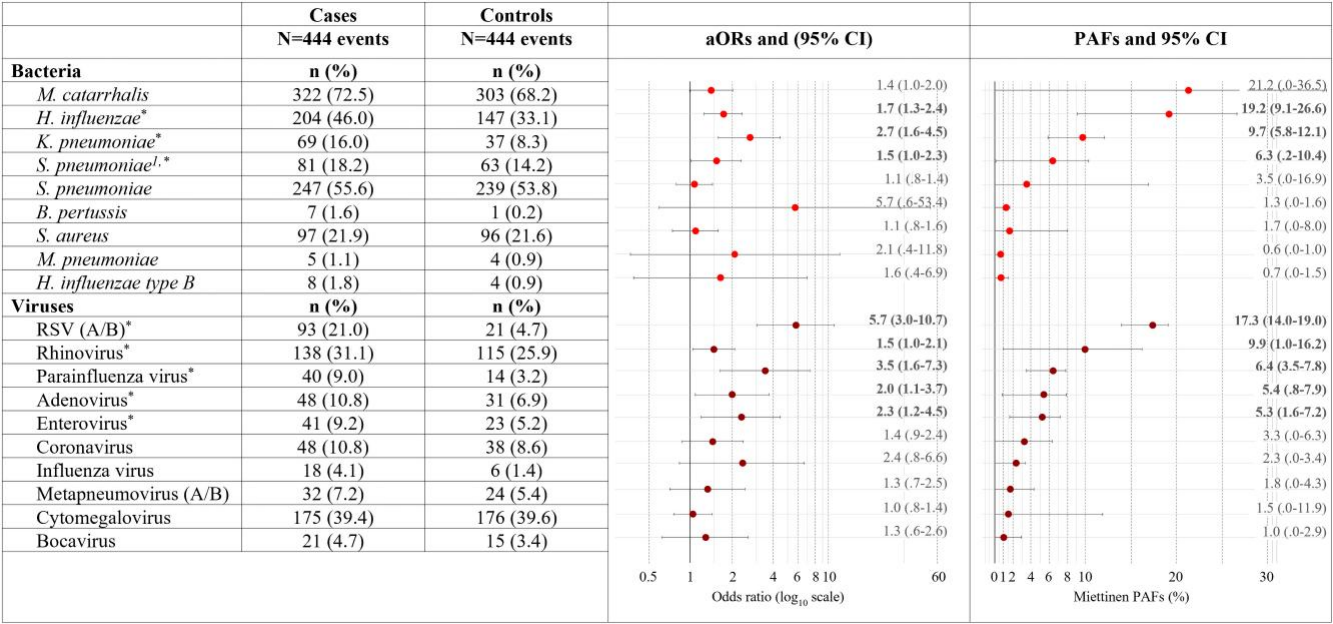
Associations between bacteria and viruses detected from nasopharyngeal (NP) specimens and lower respiratory tract infection (LRTI) during the first year of life. Adjusted odds ratios and population attributable fractions calculated for each of the NP organisms screened using the Fast-Track Diagnostics Respiratory Pathogens 33 (FTDResp33) assay for 544 participants (444 LRTI and 444 non-LRTI specimens). *Significant associations (values in bold). ^1^High-density *Streptococcus pneumoniae* (>6.9 log_10_ copies/mL). Abbreviations: aOR, adjusted odds ratio; CI, confidence interval; PAF, population attributable fraction; RSV, respiratory syncytial virus.

Using qPCR (n = 888 specimens), bacterial species associated with LRTI included *H. influenzae* (adjusted odds ratio [aOR], 1.72 [95% confidence interval {CI}, 1.25–2.37]), *Klebsiella pneumoniae* (aOR, 2.66 [95% CI, 1.59–4.46]), and high-density *S. pneumoniae* (aOR, 1.53 [95% CI, 1.01–2.32) (PAF, 6.3% [95% CI, .2–10.4) (Figure 2, Supplementary Figure 3). Age-stratified results are shown in Supplementary Figures 4–6.

Viruses associated with LRTI during the first year of life included RSV (aOR, 5.69 [95% CI, 3.03–10.69]), HRV (aOR, 1.47 [95% CI, 1.03–2.09) (PAF, 9.9% [95% CI, 1.0–16.2]), PIV (aOR, 3.46 [95% CI, 1.64–7.26), adenovirus (aOR, 1.99 [95% CI, 1.08–3.68), and enterovirus (aOR, 2.32 [95% CI, 1.20–4.46]) (Figure 2). In addition, CMV was significantly associated with LRTI among children aged 0–3 months (Supplementary Figure 4*A*). A total of 238 of 636 (37%) of specimens had ≥2 viruses detected: 148 of 323 (45.8%) in cases compared to 90 of 323 (27.9%) in controls (*P* < .001). RSV was more commonly detected in specimens from children hospitalized with LRTI compared to those with ambulatory LRTI (36.1% vs 16%, *P* < .001), whereas enteroviruses were more commonly detected in children with ambulatory, compared with hospitalized, LRTI (4% vs 13%; Supplementary Table 4). Viral detection was similar in specimens from children with a single LRTI episode compared with specimens from children experiencing >1 LRTI episode (Supplementary Table 5).

The PAF reflects the proportion of LRTI that may be attributed (alone or in combination) to a particular pathogen. For RSV, which was both prevalent and strongly associated with LRTI, the PAF was 17.3% (95% CI, 14.0%–19.0%; Figure 2). Other organisms that contributed substantially to LRTI etiology were *H. influenzae* (PAF, 19.2% [95% CI, 9.1%–26.6%]), *K. pneumoniae* (PAF, 9.7% [95% CI, 5.8%–12.1%]), and HRV (PAF, 9.9% [95% CI, 1.0%–16.2%]).

Results of qPCR in the subset of participants from whom 16S rRNA gene amplicon sequence data were available were similar to those of the full dataset (Supplementary Figure 3).

### Bacterial Community Profiles Associated With LRTI

Following quality control steps (Figure 1, Supplementary Appendix pp.19–33), a total of 323 age- and site-matched case-control sets (646 specimens) were included. The median read count per specimen was 19 283 (IQR, 13 022–25 220). Following the removal of potential contaminant ASVs (Supplementary Table 6), 826 ASVs were included, 98% of which were classifiable at the genus level.

**Figure 3.**
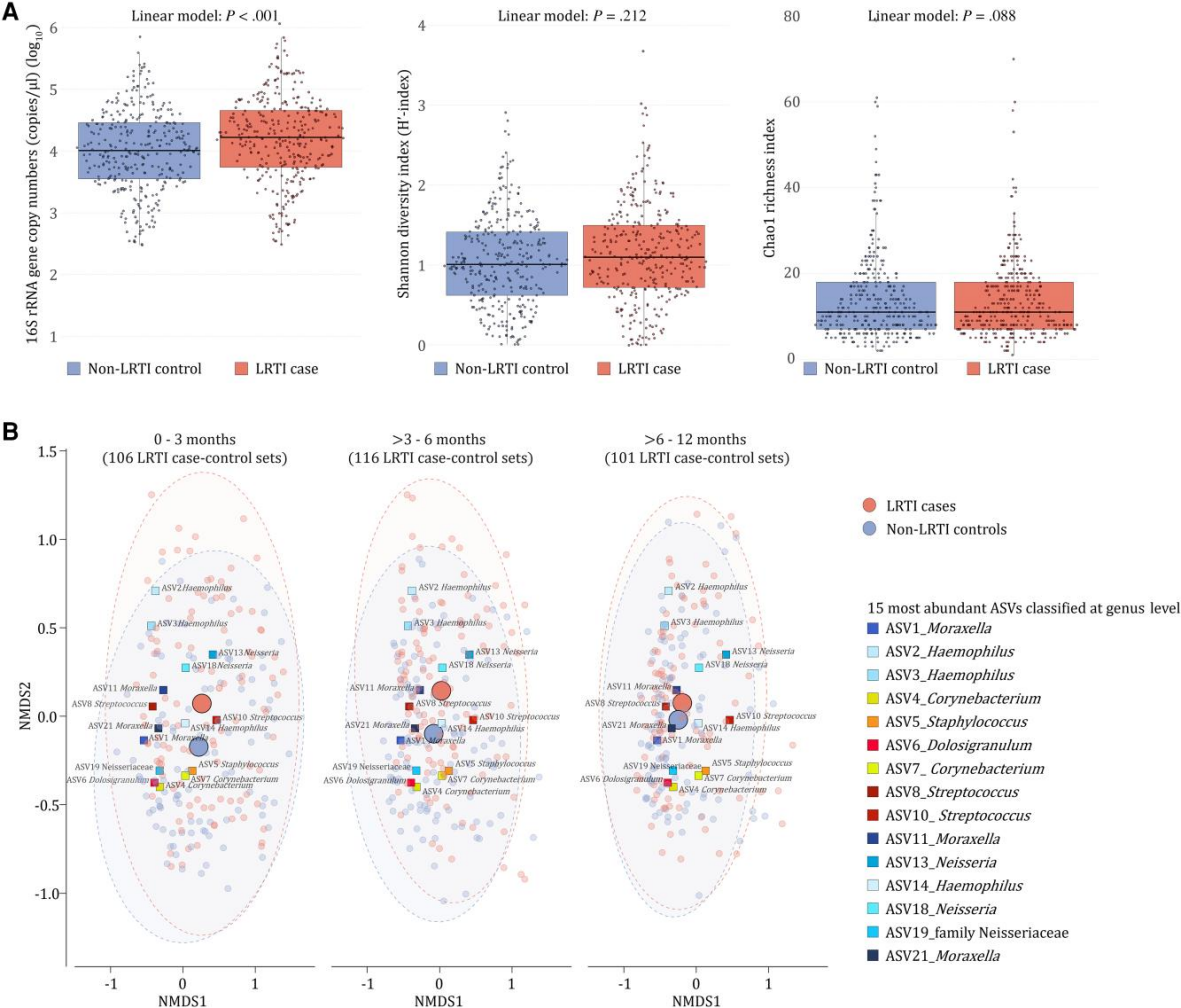
Bacterial density and diversity measured from nasopharyngeal (NP) specimens collected from lower respiratory tract infection (LRTI) cases and controls. *A*, Bacterial density (16S ribosomal RNA [rRNA] gene copies/μL) (left panel) and within-specimen bacterial diversity (Shannon diversity [middle panel] and Chao1 richness [right panel]) compared between NP specimens collected from LRTI cases and controls. *P* values are derived from linear models adjusted for specimen collection age and antibiotic therapy prior to specimen collection. Median values are presented by horizontal lines within each of the boxplots while upper and lower ranges of the boxplots represent the 75% and 25% quartiles, respectively. Maximum and minimum values, excluding outliers, are shown by whiskers. *B*, Nonmetric multidimensional scaling (NMDS) plots of Bray-Curtis dissimilarity showing loadings for the 15 most abundant amplicon sequence variants (ASVs) present in the dataset. ASVs are denoted using multicolored squares. Shades of multicolored squares are used to present phylum-level classification of each of the ASVs (Proteobacteria; Actinobacteria; Firmicutes). The 3 NMDS plots show NP specimens collected at 0–3 months (left), >3–6 months (middle), and >6–12 months (right). Small circles represent NP specimens collected from cases and controls, respectively. Large circles represent centroids of cases and controls, respectively. The number of case-control sets included in each age interval is shown at the top of each NMDS plot. Alpha bags enclosing 90% of samples are shown for cases and controls.

NP bacterial load (16S rRNA gene copy number) was higher in specimens from cases compared with controls (*P* < .0001; Figure 3*A*), and lower in specimens from infants who had antibiotic therapy prior to specimen collection, compared to no antibiotic therapy (antibiotic therapy ≤24 hours prior to collection, *P* = .004; >24 hours to ≤7 days, *P* < .001). Within-sample diversity (Shannon diversity index: *P* = .213; Chao1 index: *P* = .088) was not significantly different between cases and controls (Figure 3*A*). Higher α-diversity was observed in specimens from infants who had received antibiotic therapy compared with those who had not (Shannon *P* = .036 and Chao1 *P* = .011 for >24 hours to ≤7 days; Chao1 *P* = .026 for <24 hours). There were small but significant differences in overall NP microbial composition between cases and controls (PERMANOVA: *P* < .001, permutational multivariate analysis of dispersion: *P* <.001) (Figure 3*B*).

### Composition of the Nasopharyngeal Microbiota Varied With Age, Antibiotic Therapy, and Case Status

**Figure 4.**
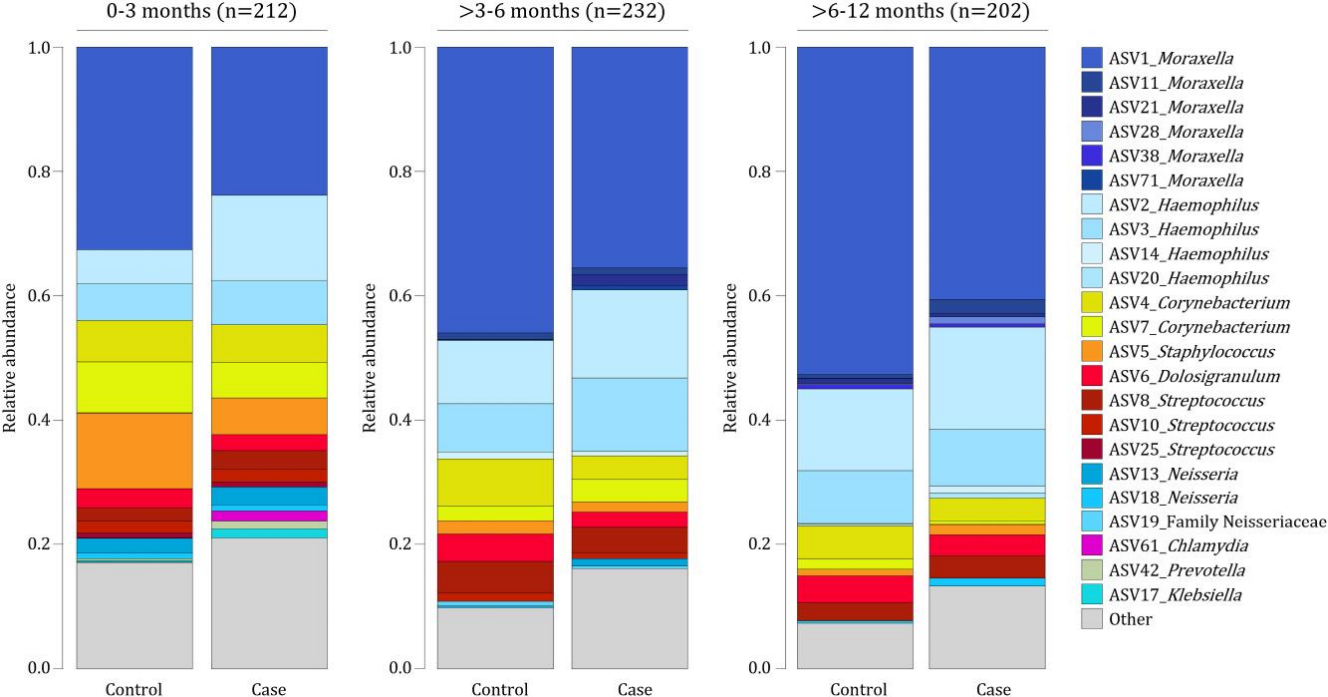
Barplots of compositional mean relative abundances of the top 15 amplicon sequence variants (ASVs) detected from nasopharyngeal specimens collected at 0–3 months (n = 212), >3–6 months (n = 232), and >6–12 months (n = 202). The 15 most abundant ASVs from each age group are shown for lower respiratory tract infection cases and controls using colors representing phylum-level classification (Proteobacteria; Actinobacteria; Firmicutes; Bacteroidetes).

Compositional mean relative abundances of the top 15 ASVs in each age category are summarized in Figure 4 and Supplementary Table 7. Participant age was negatively associated with relative abundance of *Staphylococcus*, *Corynebacterium*, and *Streptococcus* (ASV10) and positively associated with *Moraxella*, *Haemophilus*, *Dolosigranulum*, and *Streptococcus* (ASV8) (Supplementary Table 8). Antibiotic therapy prior to sampling was associated with enrichment of the anaerobes *Alloprevotella* and *Porphyromonas*, as well as *Streptococcus* ASV10 and the family Neisseriaceae (ASV19) (Supplementary Table 9).

**Figure 5.**
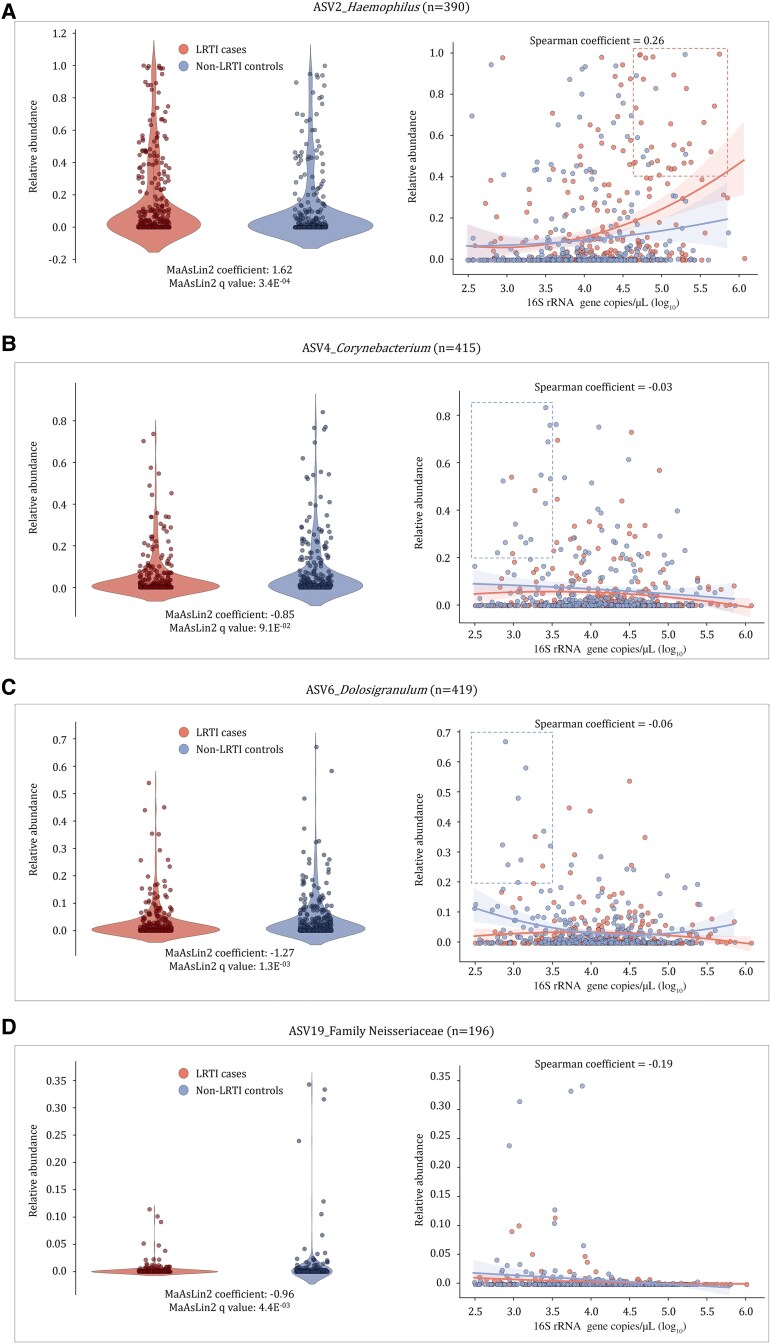
Relative abundances of amplicon sequence variants (ASVs) associated with lower respiratory tract infection (LRTI) case or control status. Relative abundances of *Haemophilus* (ASV2) (*A*), *Corynebacterium* (ASV4) (*B*), *Dolosigranulum* (ASV6) (*C*), and an unclassified ASV from the family Neisseriaceae (ASV19) (*D*) detected from nasopharyngeal (NP) specimens collected from LRTI cases and controls. Violin plots on the left of each plot show the distribution of relative abundances for each of the ASVs detected from NP specimens collected from cases and controls. Differential abundance testing results (q values and coefficients) using Microbiome Multivariable Associations with Linear Models (MaAsLin2) are shown for case-control status. ASVs with *P* values <.05 and q values <.25 were considered significantly differentially abundant. MaAsLin2 linear models were adjusted for age at specimen collection and antibiotic therapy prior to specimen collection. Scatterplots of relative abundances of each ASV plotted against bacterial load (16S ribosomal RNA [rRNA] gene copies/μL) are shown on the right of each plot. Trendlines represent the trends estimated by locally estimated scatterplot smoothing. Shaded areas represent 95% confidence intervals.

When adjusting for age and antibiotic therapy, specimens from cases had higher relative abundances of *Haemophilus* (ASV2) compared to controls (Figures 4 and 5*A*). NP specimens with both high bacterial load (>50 000 16S rRNA gene copies/μL) and high relative abundances (>40%) of *Haemophilus* (ASV2) were primarily collected from cases (77% [27/35]) (McNemar test: *P* < .0001). In contrast, *Corynebacterium* (ASV4), *Dolosigranulum* (ASV6), and an unclassified genus of the family Neisseriaceae (ASV19) were detected at significantly higher relative abundances from controls (Figure 5*B*–*D*). NP specimens with both low bacterial load (<3000 16S rRNA gene copies/μL) and high relative abundances (>20%) of *Corynebacterium* (ASV4) or *Dolosigranulum* (ASV6) were primarily collected from controls (81% [13/16], McNemar test: *P* < .0001 or 81% [9/11], McNemar test: *P* < .0001, respectively). Specimens from children experiencing >1 LRTI episode had higher relative abundances of *Haemophilus* (ASV2) and *Staphylococcus* (ASV5), and lower relative abundances of Neisseriaceae (ASV19) and *Streptococcus* (ASV10), compared to specimens from children with only a single LRTI episode (Supplementary Table 10).

**Figure 6.**
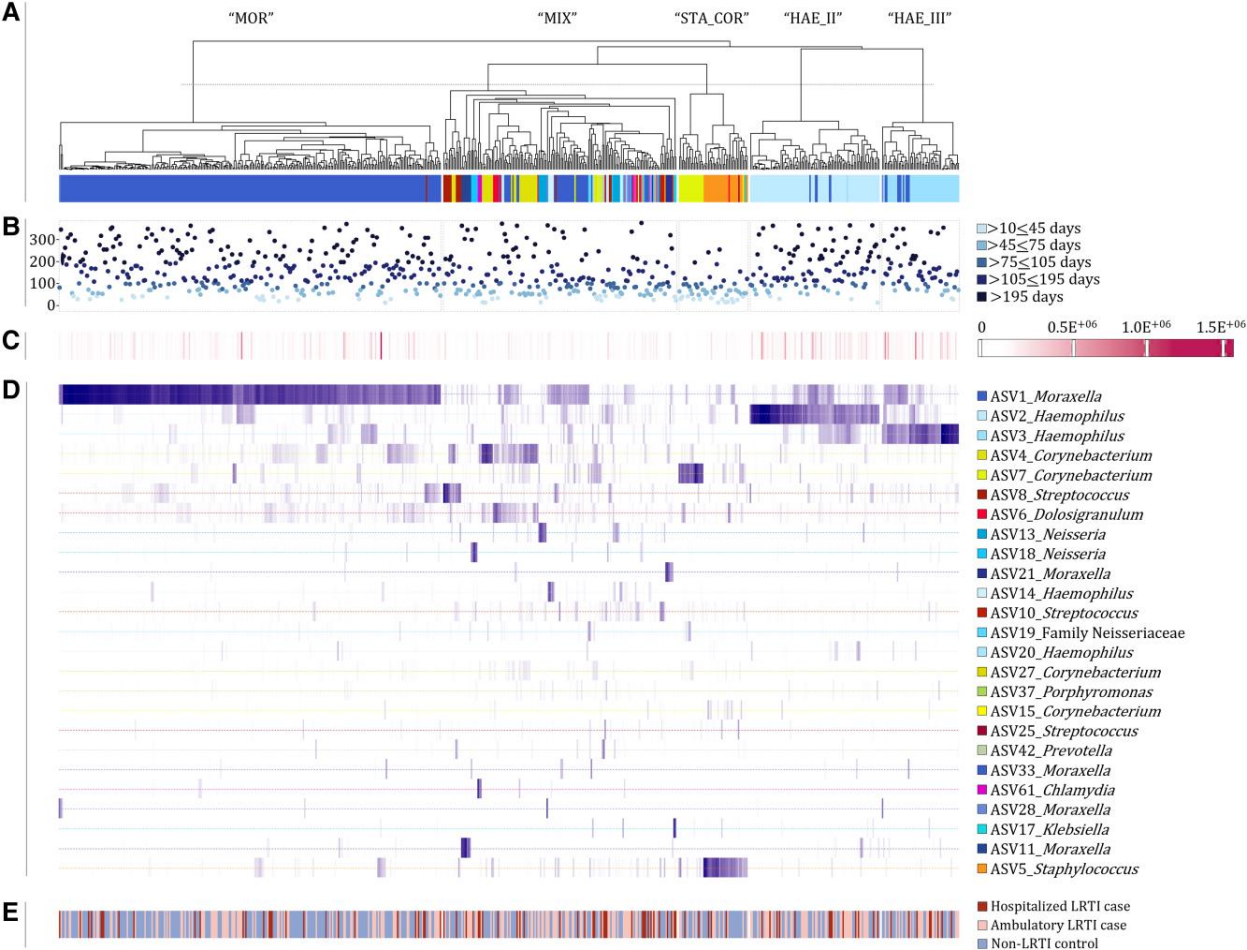
K-means clustering of relative abundances of the top 25 amplicon sequence variants (ASVs) detected from lower respiratory tract infection (LRTI) case-control nasopharyngeal (NP) specimens (n = 646). *A*, Dendogram representing unsupervised hierarchical clustering distances of NP specimens based on relative abundances of the top 25 ASVs in the dataset (n = 646). NP specimens were grouped into 5 clusters: MOR, STA_COR, HAE_II, HAE_III, and MIX. The most abundant ASVs from each specimen are shown using colors representing phylum-level classification (Actinobacteria; Bacteroidetes; Firmicutes, Proteobacteria). *B*, NP specimen collection age (in days). *C*, 16S ribosomal RNA gene amplicon copy numbers (copies/μL) measured from each NP specimen. *D*, Heatmap of relative abundances of the top 25 ASVs present in the dataset. *E*, Participant status (hospitalized case, ambulatory case, or control).

K-means clustering was used to group NP specimens (n = 646) into 5 clusters (Figure 6). The distribution of age, case status, and viral detection by cluster membership is shown in Supplementary Table 11. Compared with membership of cluster HAE_II, dominated by ASV2 (*Haemophilus*), membership of cluster STA_COR, dominated by ASV5 (*Staphylococcus*) or ASV7 (*Corynebacterium*), was negatively associated with case status (estimate −0.72, *P* = .001; Supplementary Table 12).

### Associations Between Viral Detection and Bacterial Composition

**Figure 7.**
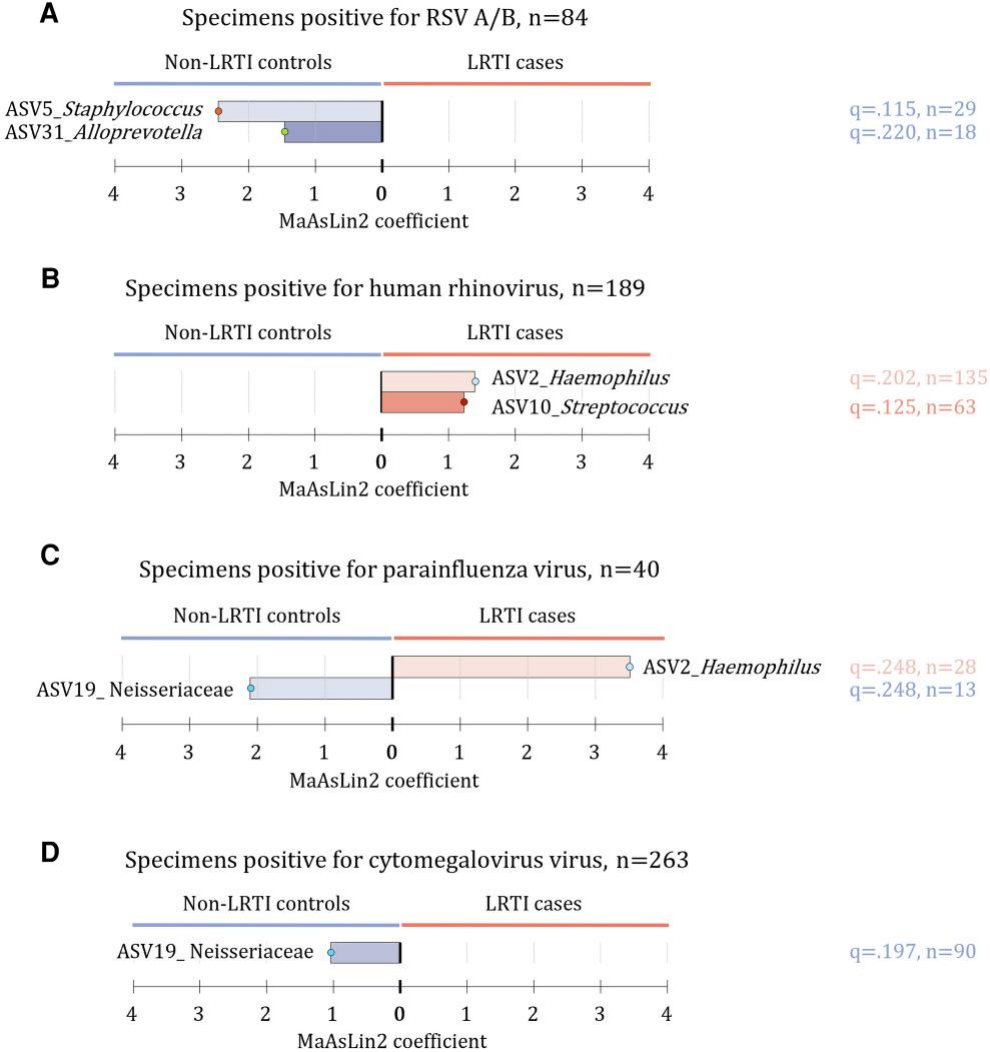
Differential abundance testing for viral and bacterial organisms detected using multiplex quantitative polymerase chain reaction (qPCR). Bacterial taxa that were associated with case status (case vs control, using linear regression models adjusted for age and antibiotic therapy prior to specimen collection) are shown for subsets of nasopharyngeal specimens positive on multiplex PCR for respiratory syncytial virus (*A*), human rhinovirus (*B*), parainfluenza virus (*C*), and cytomegalovirus (*D*). Abbreviations: ASV, amplicon sequence variant; LRTI, lower respiratory tract infection; RSV, respiratory syncytial virus.

To explore whether the microbiota was associated with development of LRTI in infants infected with a particular respiratory virus, we investigated which bacterial taxa were associated with case status among infants infected with specific viruses (including cases and controls). Among the subset of infants in whom RSV was detected (68 cases and 16 controls), we observed significantly higher relative abundances of *Staphylococcus* spp (ASV5) and *Alloprevotella* spp (ASV31) in controls when compared to cases (Figure 7*A*). Among infants in whom HRV was detected (103 cases and 86 controls), we observed higher abundances of *Moraxella* spp (ASV1) in controls than cases, and higher abundances of *Haemophilus* spp (ASV2) and *Streptococcus* spp (ASV10) in cases (Figure 7*B*). Similarly, in infants with PIV (30 cases and 10 controls), *Haemophilus* spp (ASV2) relative abundance was higher among cases compared to controls. An unclassified genus within the family Neisseriaceae had higher relative abundance in controls compared to cases among infants infected with PIV (Figure 7*C*) or CMV (133 cases and 130 controls) (Figure 7*D*).

We also explored whether bacterial taxa were associated with detection of viruses, stratified by case-control status (Table 2). In controls, CMV was negatively associated with the relative abundance of *Staphylococcus* (ASV5), *Dolosigranulum* (ASV6), and *Corynebacterium* (ASV4), whereas HRV was negatively associated with *Staphylococcus* (ASV5), *Corynebacterium* (ASV7), and *Streptococcus* (ASV10). These associations were not observed in children with LRTI; however, HRV was positively associated with *Haemophilus* (ASV2) in both cases and controls.

**Table 2.** Association Between Selected Viral Targets (Detected by Quantitative Real-Time Polymerase Chain Reaction Panel) and Bacterial Taxa (Amplicon Sequence Variant From 16S Ribosomal RNA Gene Amplicon Sequencing)

Bacterial ASV and Virus Pair	Association in Controls	Association in Cases
	Regression Coefficient (*P* Value; q Value)	
(ASV6) *Dolosigranulum pigrum* | CMV	**−1.00 (.03; .06)**	0.10 (.85; .94)
(ASV4) *Corynebacterium* | CMV	**−1.10 (.04; .07)**	0.41 (.37; .59)
(ASV5) *Staphylococcus* | CMV	**−1.47 (<.01; .01)**	−0.13 (.77; .89)
(ASV3) *Haemophilus* | CMV	**1.26 (.02; .04)**	0.50 (.26; .49)
(ASV7) *Corynebacterium* | Rhinovirus	**−0.92 (.04; .07)**	−0.02 (.96; .96)
(ASV5) *Staphylococcus* | Rhinovirus	**−1.8 (<.01; <.01)**	−0.71 (.13; .25)
(ASV10) *Streptococcus* | Rhinovirus	**−0.90 (.05; .08)**	0.11 (.80; .85)
(ASV2) *Haemophilus* | Rhinovirus	**1.17 (.04; .08)**	**1.09 (.05; .12)**
(ASV1) *Moraxella* | Rhinovirus	**1.35 (<.01; .02)**	0.51 (.31; .41)
(ASV36) *Johnsonella* | Rhinovirus	0.32 (.35; .46)	**1.0 (<.01; <.01)**
(ASV19) Family Neisseriaceae | RSV_A_B	0.40 (.67; .79)	**0.93 (.03; .08)**
(ASV40) *Gemella* | RSV_A_B	0.54 (.54; .79)	**−0.91 (.03; .08)**

Logistic regression (MaAsLin2) model included age and antibiotic treatment. Only significant results (*P* < .05) are shown. Significant results (*P* < .05 and q < .25) are shown in bold.

Abbreviations: ASV, amplicon sequence variant; CMV, cytomegalovirus; RSV, respiratory syncytial virus.

## DISCUSSION

We investigated associations between NP bacterial community profiles, viral infection, and LRTI in South African infants. The viruses RSV, HRV, PIV, adenovirus, and enterovirus and the bacteria *H. influenzae*, *K. pneumoniae*, and *S. pneumoniae* were associated with LRTI during the first year of life. LRTI was associated with lower relative abundances of the commensal taxa *Corynebacterium*, *Dolosigranulum*, and an unclassified genus within the family Neisseriaceae. Among children with HRV detected, *Haemophilus* or *Streptococcus* was positively associated with LRTI, and in those with PIV infection, *Haemophilus* was positively associated with LRTI. In contrast, among children with RSV detected, *Staphylococcus* or *Alloprevotella* were negatively associated with LRTI. In children without LRTI, CMV or HRV detection was associated with lower relative abundance of *Staphylococcus* and *Corynebacterium*, while CMV infection was also associated with lower relative abundance of *Dolosigranulum*.

Our findings of a positive association of LRTI with *Haemophilus* and a negative association with *Corynebacterium* and *Dolosigranulum* are strikingly similar to those observed in children with upper or lower respiratory infections in the United States [19], Australia [20], Europe [21], and Botswana [22]. This finding therefore represents a conserved phenotype associated with respiratory infection across both high- and middle-income settings. Negative interactions between beneficial microbes and pathobionts (commensal microbiome organisms that can become pathogenic under certain environmental circumstances or following genetic changes) may explain the protective role of genera like *Corynebacterium*, which can inhibit *S. pneumoniae* in vitro and in a mouse colonization model [23]. The combination of high bacterial load and high relative abundance of *Haemophilus* was strongly associated with LRTI, while the combination of low bacterial load and high relative abundance of *Corynebacterium* or *Dolosigranulum* was associated with health. This supports the hypothesis that translocation of large numbers of pathobionts from the nasopharynx to the lower respiratory tract may be key to pathogenesis of LRTI. We did not find evidence that the overall bacterial diversity within the NP was associated with respiratory infection, in contrast to previous findings in acute otitis media [24] and chronic rhinosinusitis [25].

In keeping with our previous findings [11, 26], we detected significant associations between LRTI and detection of viruses (RSV, HRV, PIV, and adenovirus) and bacteria (*H. influenzae*, *S. pneumoniae*, and *K. pneumoniae*). Our findings are also similar to those previously reported in the multicenter Pneumonia Etiology Research for Child Health (PERCH) study [27]. Although we excluded congenital pneumonia cases from our analysis, we identified an association between CMV detection from the NP and LRTI in the first 3 months of life. CMV viremia has been described in infants with hypoxic pneumonia [28], although whether CMV contributes to respiratory illness or whether illness triggers CMV viremia is unclear. Autopsy studies have also identified CMV as an important contributory cause to death in children aged <5 years [29].

RSV contributed to a substantial proportion of cases (PAF, 17%). In contrast, interpretation of the contribution of the highly prevalent microbes *H. influenzae*, *Moraxella catarrhalis*, and HRV to LRTI is less straightforward. These microbes were only weakly associated with LRTI, and were commonly detected in healthy children, suggesting that interactions between these microbes and pathobionts or host factors may be required for progression to LRTI. We therefore explored interactions between viruses and bacteria.

Among children in whom RSV was detected, the relative abundance of *Staphylococcus* or *Alloprevotella* was lower in cases compared with controls. Several reports have identified lower prevalence or abundance of *Staphylococcus* in children with RSV infection, compared with controls [5, 30]. Neutrophilic inflammation in the respiratory tract has been shown to predict symptomatic RSV disease [31], and *S. aureus* nasal colonization impairs neutrophil recruitment [32], suggesting that *S. aureus* colonization may reduce the risk of symptomatic RSV infection.

Among those infants infected with HRV or PIV, *Haemophilus* relative abundance was higher among cases compared with controls. This is in keeping with the hypothesis that for viruses with lower pathogenicity, coinfection with bacteria, particularly *H. influenzae*, is important in driving progression to LRTI. Unlike previous reports [5, 30], we did not identify an association between RSV LRTI and *H. influenzae* (ASVs 2 or 3) or *S. pneumoniae* infection.

The role of *Moraxella* remains unclear. *Moraxella* ASV1 was the most abundant ASV detected, in keeping with our previous finding [33]. qPCR targeting *M. catarrhalis* showed a positive association with LRTI; however, the genus *Moraxella* was not associated with LRTI overall, and, in children with HRV infection, was associated with control status. ASV1 also includes the understudied species *Moraxella nonliquefasciens*, and it is possible that these 2 species of *Moraxella* are differentially associated with LRTI.

Our finding that, in control children, CMV infection was associated with reduced relative abundance of gram-positive taxa, including the health-associated *Dolosigranulum* and *Corynebacterium*, as well as *Staphylococcus*, is intriguing. CMV infection or reactivation may be a marker of impaired immunity and has been associated with subsequent increased risk of tuberculosis in this cohort [34]. CMV has been associated with alterations of the gut microbiota, which mediates the risk of allergic sensitization [35]. The lower relative abundance of health-associated gram-positive bacteria in children with CMV infection may reflect CMV-impaired local immunity or may increase the risk of CMV infection or reactivation and may in turn negatively impact mucosal immunity to pathogens, such as tuberculosis.

Our study has several key strengths, including a robust birth cohort study design that reduces the risk of bias in identifying cases and controls, large sample size, and detection of viruses and bacteria using highly multiplexed qPCR as well as 16S rRNA amplicon sequencing. There are several limitations to our study. First, although this is among the largest studies of its kind, our ability to study viral–bacterial interactions was limited for less prevalent viruses. Second, due to the cross-sectional study design, causal inferences are not possible. Third, while the NP niche is a reasonable proxy for the lower respiratory tract [36], we were unable to directly sample the lung. A limitation of short-read 16S rRNA amplicon sequencing is taxonomic resolution to the genus and not species level. Finally, host responses are required to better understand host–microbe interactions in LRTI.

Conserved microbial patterns distinguishing respiratory infection from health in children across several continents strongly suggest that the identified microbes play a role in maintaining respiratory health or driving disease. Whether these patterns are causal is unknown; studies exploring whether modulation of the respiratory microbiota can influence respiratory infections are needed.

## Supplementary Material

ciaf184_Supplementary_Data

## Supplementary Data

Supplementary materials are available at *Clinical Infectious Diseases* online. Consisting of data provided by the authors to benefit the reader, the posted materials are not copyedited and are the sole responsibility of the authors, so questions or comments should be addressed to the corresponding author.

## Notes

***Author contributions*.** Conception and design of the parent study (Drakenstein Child Health Study [DCHS]) and the current study: H. J. Z. and M. P. N. Funding acquisition: M. P. N., W. C. N., and H. J. Z. FTDResp33 data generation and quality control: F. S. D., S. A., F. P., V. A., and L. A. T. W. 16S rRNA amplicon sequencing training: S. H. M. 16S rRNA amplicon sequencing data generation: S. C.-W. 16S rRNA amplicon sequencing data quality control: S. C.-W. and S. G.-L. Bioinformatics processing: K. S. M. Data analysis and interpretation: M. P. N., S. C.-W., L. W., F. S. D., S. G.-L., L. H., and Y. X. Initial draft of the manuscript: S. C.-W. Major contributions to writing of the manuscript: S. C.-W. and M. P. N. Manuscript revisions: S. C.-W., L. W., L. H., Y. X., H. J. Z., and M. P. N. All authors read and approved the final manuscript.

***Acknowledgments*.** The authors thank Drakenstein Child Health Study (DCHS) participants and their families, as well as the DCHS clinical, laboratory, and data team staff. The authors thank the staff of the Western Cape Government Health Department at Paarl Hospital and at the clinics for support of the study. The authors also acknowledge facilities provided by the University of Cape Town's Information and Communication Technology Services (ICTS) High Performance Computing Team (http://hpc.uct.ac.za). The authors thank Prof. Debby Bogaert's team at the Centre for Inflammation Research, University of Edinburgh, for guidance related to the qPCR protocol.

***Data and code availability*.** The qPCR data are available at https://github.com/xy-repo/Drakenstein-Nested-Case-Control. The 16S rRNA gene sequencing data supporting the conclusions of this article will be made available in the National Center for Biotechnology Information Sequence Read Archive. Metadata included in this study are available at Drakenstein-Nested-Case-Control/raw_data at main xy-repo/Drakenstein-Nested-Case-Control. This manuscript reports original code for MaAsLin2 analyses, which is available at https://github.com/xy-repo/Drakenstein-Nested-Case-Control.

***Disclaimer.*** The funders had no role in the design of the study and collection, analysis and interpretation of the data, writing of the manuscript, or the decision to publish this report.

***Financial support*.** This work was supported by an H3Africa U01 award from the US National Institutes of Health (grant number 1U01AI110466-01A1); the Gates Foundation (grant numbers OPP1017641 and OPP1017579); the National Research Foundation of South Africa; and the South African Medical Research Council. S. C.-W. was funded by the Gates Foundation (grant number OPP1017641), the National Research Foundation of South Africa, and the L’Oréal-UNESCO For Women in Science program (South African Young Talents Award). S. H. M. and W. C. N. were supported by the Gates Foundation. M. P. N. was supported by an Australian National Health and Medical Research Council Investigator Grant (grant number APP1174455). H. J. Z. was supported by the South African Medical Research Council.

***Potential conflicts of interest**.* The authors: No potential conflicts. All authors have submitted the ICMJE Form for Disclosure of Potential Conflicts of Interest.
