# Supplementary material for: Nasopharyngeal Microbiota in South African Infants With Lower Respiratory Tract Infection: A Nested Case-Control Study of the Drakenstein Child Health Study
Source: Clin Infect Dis. 2025 Apr 17;81(6):e668–79. doi: 10.1093/cid/ciaf184 (PMC13375580; doi:10.1093/cid/ciaf184)
Supplement: ciaf184_Supplementary_Data [file ciaf184_supplementary_data.docx]

Supplementary appendix for

**Nasopharyngeal microbiota in South African infants with lower respiratory tract infection: a nested case-control study of the Drakenstein Child Health Study**

Shantelle Claassen-Weitz, Yao Xia, Lesley Workman^,^ Luke Hannan, Sugnet Gardner-Lubbe, Kilaza S Mwaikono, Stephanie Harris Mounaud, William C. Nierman, Samantha Africa, Fadheela Patel, Felix Sizwe Dube, Veronica Allen, Lemese Ah Tow Edries, Heather J. Zar, Mark P. Nicol^*^

Correspondence to: mark.nicol@uwa.edu.au

**Supplementary Methods:**

1. Study design and enrolment p. 2
2. Demographic and clinical data p. 3
3. LRTI case and non-LRTI control matching (Figure S1) pp. 3-4
4. Laboratory procedures

- *Nucleic acid extraction* p. 4
- *Sequencing controls (Figure S2)* pp. 4-5
- *16S rRNA gene amplicon library preparation and sequencing* pp. 5-7

1. Bioinformatics workflow, in-silico quality control, and statistical analysis

- *Bioinformatics workflow* p. 7
- *In-silico quality control approach for* *short read 16S rRNA gene amplicon sequencing data* p. 8

**Supplementary Results:**

1. Table S1. Characteristics of DCHS study population included in the analysis. pp. 9-10
2. Table S2. Prevalence of viruses in specimens collected prior to LRTI diagnosis vs

at or after LRTI diagnosis. p. 11

1. Table S3. Clinical symptoms in children whose specimens were collected

prior to LRTI diagnosis vs specimens collected at or after LRTI diagnosis. p. 12

1. Table S4. Prevalence of viral and bacterial targets detected by qPCR in specimens

collected at time of event, stratified by hospitalized or ambulatory LRTI. p. 13

1. Figure S3-S6. Molecular (qPCR) detection of nasopharyngeal pathogens commonly associated with LRTI. pp. 14-17
2. Table S5. Prevalence of viruses in children with a single LRTI compared to p. 18

children with ≥ 1 LRTI.

1. In-silico quality control of short read 16S ribosomal ribonucleic acid (rRNA) gene amplicon sequencing data: pp. 19-33

- *Sequencing reproducibility*
- *Identification and removal of potential contaminant amplicon*

*sequence variants (ASVs) (Table S6)*

1. Table S7. Compositional mean relative abundances of the top 15 amplicon sequence variants (ASVs) in each age category (0-3 months, >3-6 months, >6-12 months). p. 34
2. Table S8. Differential abundance testing for age at specimen collection. p. 35
3. Table S9. Differential abundance testing for commencement of antibiotic therapy

prior to specimen collection. p. 36

1. Table S10. Bacterial taxa which were differentially abundant in children with

more than one LRTI episode compared with those with only one LRTI episode. p. 37

1. Table S11. Five clusters based on the relative abundances of the top 25 ASVs

in the dataset. p. 38

1. Table S12. Associations between cluster membership and LRTI case status,

controlling for age, bacterial load, and viral abundance p. 39

**Supplementary References** p. 40

**Supplementary Methods**

**Study design and enrolment:**

We conducted a case-control study of infants enrolled in the Drakenstein Child Health Study (DCHS), a birth cohort study in a peri-urban area in South Africa [1]. Pregnant women >18 years of age residing in a peri-urban setting (Paarl, outside Cape Town, SA) provided written informed consent at enrolment (20-28 weeks gestation) and reconsented annually [1]. Enrolment (March 5, 2012 to March 31, 2015) and primary health care took place at public sector primary healthcare clinics, characterised by a strong primary health care program including antenatal services, immunization, and prevention of mother-to-child HIV transmission (PMTCT), as previously described [1].

**Demographic and clinical data collection:**

Antenatal ultrasound data from the second trimester was used to calculate gestational age at delivery. Infants born at <37 weeks gestation were classified as premature. Weight-for-age z- (WAZ) scores at birth were calculated using the revised Fenton preterm growth charts [2,3]. Maternal smoking was self-reported antenatally and 10 weeks postnatally. Maternal HIV infection status was established in pregnancy and antiretroviral therapy (ART) was provided for all HIV-infected mothers according to national guidelines [4,5]. HIV-exposed infants were tested for HIV as per national guidelines [4,5]. HIV-exposed infants who tested negative for HIV were classified as HIV-exposed, uninfected. Nasopharyngeal (NP) specimen collection date and date of birth were used to determine specimen collection age. NP specimen collection date and date of antibiotic use were used to determine duration of antibiotic use at specimen collection. Variables representing socio-economic status (SES) included household density, household monthly income, parental employment, and maternal education. Immunizations were recorded at routine immunization visits.

**LRTI case and non-LRTI control matching:**

NP specimens were collected 2-weekly from infants during the first year of life (<365 +14 days) (Figure S1 A). NP specimens from LRTI cases and non-LRTI controls were matched 1:1 by birth date (max age difference: +/-14 days) and study site (TC Newman and Mbekweni). NP specimens from LRTI cases were only included as case specimens if collected within +/-14 days of a LRTI episode (Figure S1 B). Recurring case specimens collected >28 days apart were considered eligible for inclusion as case specimens in independent case-control sets (Figure S1 C). Non-LRTI NP specimens from LRTI cases were eligible for inclusion as non-LRTI control specimens if the LRTI episode occurred >28 days prior to and after collection of the non-LRTI control specimen (Figure S1 D).


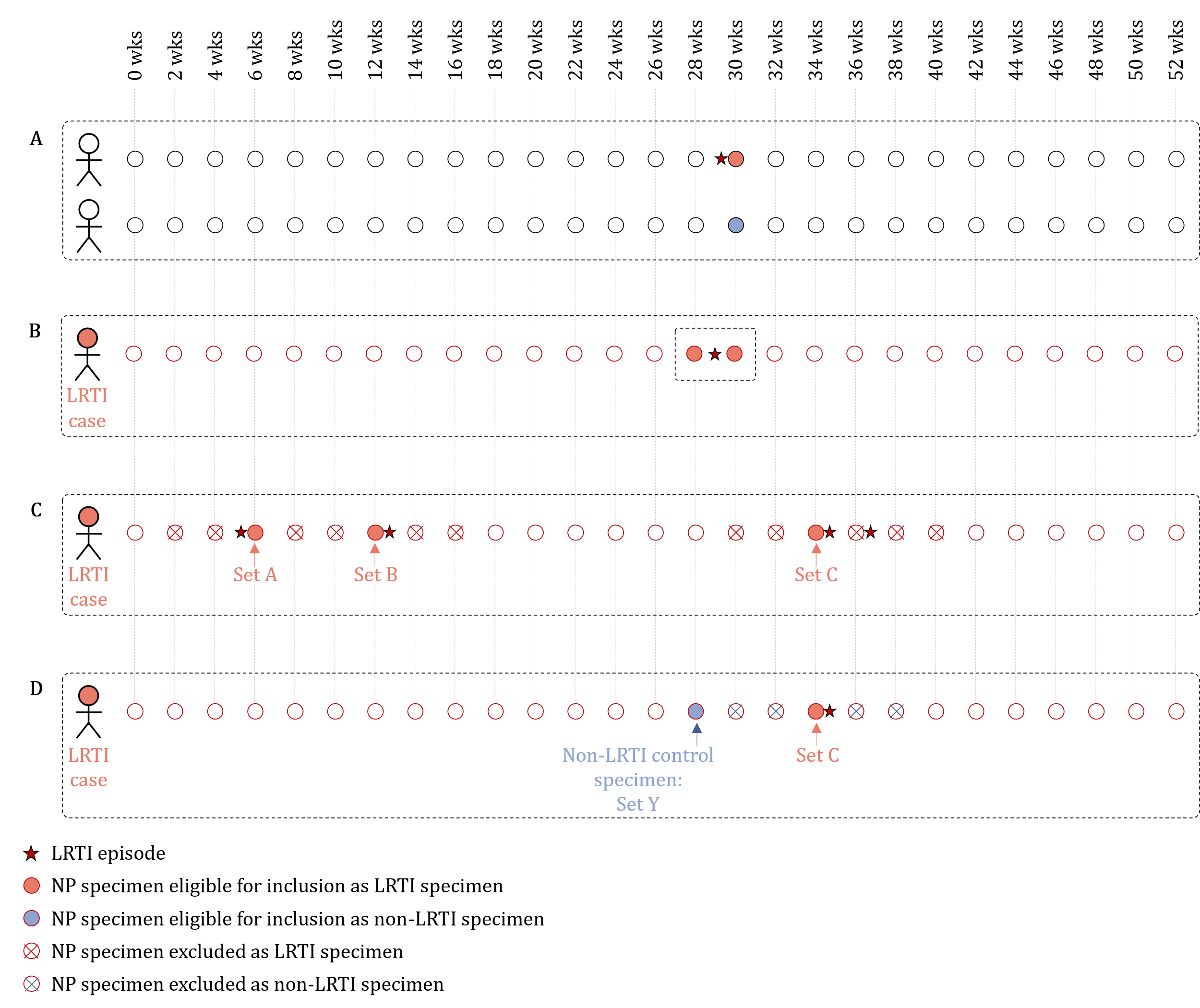


**Figure S1** **Matching LRTI cases to non-LRTI controls.**

A) Date of birth and study site were used to perform 1:1 matching of NP specimens collected from LRTI cases and non-LRTI controls. B) Only NP specimens collected within +/- 14 days of a LRTI episode were included as LRTI case specimens. C) Recurring LRTI case specimens from a single participant were eligible for inclusion as LRTI cases specimens in more than one case-control sets if the recurring specimens were collected >28 days apart. D) Non-LRTI NP specimens collected >28 days prior to and after a LRTI event were eligible for inclusion as non-LRTI control specimens.

Demographic and clinical data of a participant included as a LRTI case in more than one case-control set (Figure S1 C) only featured once in the summary demographics table. Similarly, the latter was applied to a participant included as a non-LRTI control in more than one case-control set. If a participant was included as a LRTI case in one case-control set and non-LRTI control another (Figure S1 D), demographic and clinical data of the participant would be included in the summary demographics table as data from case-control participants, individually.

**Laboratory procedures:**

*Nucleic acid extraction*

Nucleic acid extraction was performed on 400 µl of homogenised NP specimen. Homogenised NP specimen was transferred to ZR BashingBead™ Lysis Tubes containing 0.5 mm bashing beads (catalogue no. ZR S6002-50, Zymo Research Corp., Irvine, CA, USA). Mechanical lysis was performed at 50 Hz for 5 min using the TissueLyser LT™ (Qiagen, FRITSCH GmbH, Idar-Oberstein, Germany). The lysate was centrifuged at 10,000 rpm for 1 min and 250 μl of the supernatant loaded onto the QIAsymphony® SP instrument (Qiagen, Hombrechtikon, Switzerland) for DNA extraction. We used the DSP Virus/Pathogen Mini Kit® (catalogue no. 937036, Qiagen GmbH, Hilden, Germany) to carry out automated extractions of DNA with the elution volume set to 60 μl.

*Sequencing controls*

Each sequencing run consisted of four 96-well plates (384 reactions). Sequencing controls (per 96-well plate) consisted of a microbial mock community control [1-in-10 fold dilutions of microbial mock community DNA controls: HM-783D (BEI Resources, NIAID, NIH as part of the Human Microbiome Project, VA, USA) (“BEI-DNA”) and/or ZymoBIOMICS™ Microbial Community DNA Standard (catalogue no. D6305, Zymo Research Corp., Irvine, CA, United States) (“Zymobiomics-DNA”)]; a randomly selected NP specimen for repeat amplification and sequencing within each run (“within-run repeat”); a randomly selected NP specimen for repeat amplification and sequencing between different runs (“between-run repeat”); and a no template control (NTC) [neat PrimeStore® Molecular Transport medium (Longhorn Vaccines & Diagnostics, MD, USA)][6] (Figure S2)**.**


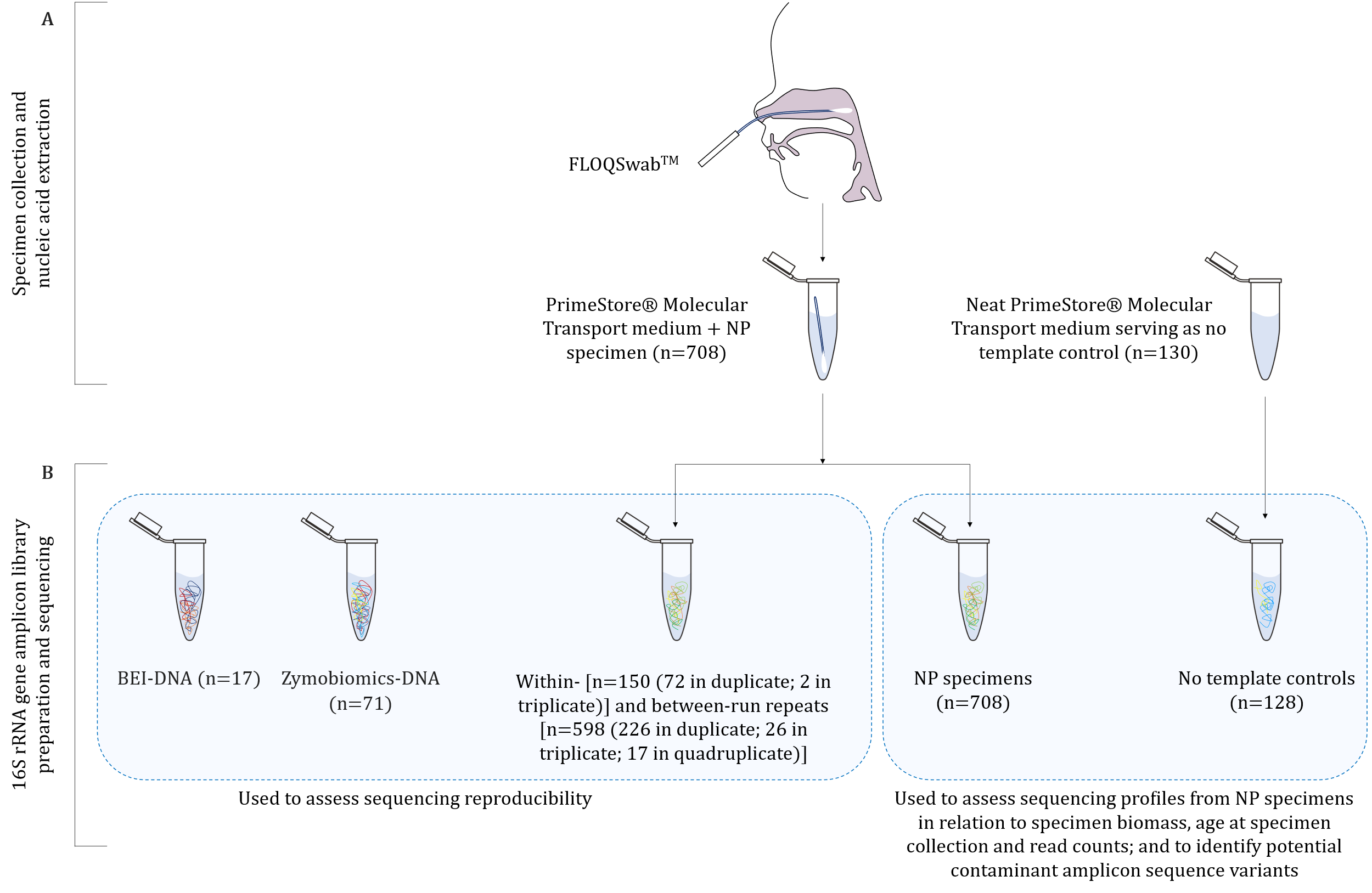


**Figure S2** **Nasopharyngeal (NP) specimens and sequencing controls processed via 16S rRNA gene amplicon library preparation and sequencing.**

A) Collection of NP specimens (FLOQSwab^TM^, Copan Diagnostics, CA, USA) suspended in PrimeStore® Molecular Transport medium (Longhorn Vaccines & Diagnostics, MD, USA) and no template controls (NTCs) (neat PrimeStore® Molecular Transport medium). Automated nucleic acid extraction was performed on the QIAsymphony® SP instrument (Qiagen, Hombrechtikon, Switzerland) using the DSP Virus/Pathogen Mini Kit® (catalogue no. 937036, Qiagen GmbH, Hilden, Germany). B) 1-in-10 fold dilutions of microbial mock community DNA controls [HM-783D (BEI Resources, NIAID, NIH as part of the Human Microbiome Project, VA, USA) (“BEI-DNA”) and/or ZymoBIOMICS™ Microbial Community DNA Standard (catalogue no. D6305, Zymo Research Corp., Irvine, CA, United States) (“Zymobiomics-DNA”)], nucleic acid extracts from NP specimens randomly selected for repeat amplification and sequencing (“within-run repeats”), nucleic acid extracts from NP specimens randomly selected from a previous sequencing run for repeat amplification and sequencing (“between-run repeats”), and nucleic acid extracts from NP specimens and NTCs were included during 16S rRNA gene amplicon library preparation and sequencing.

*16S rRNA gene amplicon library preparation and sequencing*

We measured total bacterial load (16S rRNA gene copies/μl) from nucleic acid extracted from NP specimens and sequencing controls using a previously described qPCR method targeting the 16S rRNA gene [7].

We performed a two-step amplification of the V4 hypervariable region of the 16S rRNA gene using 7 µl of nucleic acid (step 1) and 7 µl of amplicon as template (step 2).[8] PCR conditions and modified primers used in the two-step amplification approach have previously been published [8,9]. Next, we purified amplicons using Agencourt® AMPure® XP PCR Purification beads (catalogue no. A63881, Beckman Coulter, CA, USA) at a 0.65:1 (bead:amplicon) ratio [8]. Purified amplicons were verified via agarose gel electrophoresis (GloMax®-Multi Detection System, Promega Corporation, Madison, WI, USA) and quantified via double stranded DNA (dsDNA) detection (QuantiFluor® dsDNA System, catalogue no. E2670, Promega Corporation, Madison, WI, USA).

For each run (384 reactions), we pooled purified short fragment 16S rRNA gene amplicons at equimolar concentration (70 ng), whereafter we performed another round of purification using Agencourt® AMPure® XP PCR Purification beads at a 1:1 (bead:amplicon) ratio. We quantified the purified pool using the Qubit® Fluorometer (Invitrogen, Life Technologies, CA, USA) and Qubit™ dsDNA BR Assay Kit (catalogue no. Q32850, Invitrogen, Life Technologies, CA, USA). Gel electrophoresis was applied to a total of 7,000 ng of purified pooled library [8], whereafter the excised pooled library was purified (QIAquick Gel Extraction kit, QIAgen, MA, USA). We determined the pooled library fragment size using the Agilent DNA 1000 Kit (Agilent Technologies, Santa Clara, CA, USA). Quantification was performed using the KAPA Library Quantification Kit (catalogue no. KK4844, KAPA Biosystems, Boston, MA, USA).

Library loading concentrations ranged between 5.5 pM and 7 pM per library across respective sequencing runs to obtain optimal flow cell loading concentrations. Library dilution and denaturing steps have been described elsewhere [10,11]. We loaded denatured libraries containing a 15% denatured PhiX Control v3 spike-in (catalogue no. FC-110-3001, Illumina, San Diego, CA, USA) onto the MiSeq Reagent Kit v3 (600-cycle) (Illumina, San Diego, CA, USA). We loaded 3.4 µl of each 100 µM custom sequencing primers (Integrated DNA technologies, Coralville, IA, USA) [sequencing primer read 1: 5’-TATGGTAATTGTGTGCCAGCHGCYGCGGTAA-3’, sequencing primer read 2: 5’-AGTCAGTCAGCCGGACTACHVGGGTWTCTAAT-3’ and index sequence: 5’-ATTAGAWACCCBDGTAGTCCGGCTGACTGACT-3] and performed sequencing on the Illumina^®^ MiSeq™ platform.

**Bioinformatics workflow, in-silico quality control, and statistical analysis:**

*Bioinformatics workflow*

We assessed the quality of demultiplexed paired-end reads using FastQC [12] and MultiQC [13]. Thereafter, the DADA2 pipeline [14] (wrapped in the Nextflow algorithm) [15] was used to filter and trim reads, infer amplicon sequence variants (ASVs), and assign taxonomy to ASVs. We trimmed and truncated forward reads at 24 and 248 bases, and reverse reads at 25 and 235 bases. We further truncated reads at the first instance of a quality score <2, trimmed reads containing >2 expected errors, and discarded reads with any ambiguous bases. We applied a minimum read length of 250 bases after trimming and truncation.

We applied unsupervised machine-learning to estimate a parametric error model on 100 million sequences (forward and reverse reads separately). We dereplicated sequencing reads and inferred ASVs for each sample using the DADA2 sample inference algorithm (pseudo-pooling) and the estimated error model. We merged denoised sequences using the inferred forward and reverse reads (length of overlap between forward and reverse reads was set to 20). We allowed for no mismatches in the overlap region. We identified and removed chimeric sequences via the removeBimeraDenovo function (method = “consensus”).

We assigned taxonomy to each of the ASVs using the RDP [16] classifier implementation for DADA2 [17] (taxassignment = ‘rdp’) and SILVA version 138 (reference = SILVA 138; species = SILVA 138) [18]. We excluded ASVs classified as Eukaryota and ASVs with unassigned taxonomy at Kingdom-level for downstream analyses.

*In-silico quality control approach for short read 16S rRNA gene amplicon sequencing data*

We used R software version 4.1.2 [19] and RStudio version 2021.09.2 [20]. We validated library preparation and sequencing steps by comparing manufacturers’ specified compositions of bacterial mock community DNA controls to those generated in our laboratory. We investigated sequencing reproducibility of NP specimens randomly selected for repeat sequencing within and between runs (“within- and between-run repeats”). Sequencing reproducibility of within- and between-run repeats was investigated in relation to specimen biomass (16S rRNA gene copies/µl), demographic data (participant age at specimen collection) and read counts, as previously described [6]. We determined how bacterial community profiles from NP specimens compared to those from NTCs in relation to specimen biomass, age at specimen collection and read counts, as previously described [6]. We used 1) sequencing reproducibility and 2) parallels between NP specimens and NTCs in relation to biomass, age at specimen collection, and read counts, to set cut-offs for excluding NP specimens from downstream analyses.

We identified and removed potential “contaminant ASVs” from the dataset by implementing the isContaminant function [21] and a combination of the “frequency- and prevalence-based methods” offered by the *decontam* package in R (method=“combined”) [21]. The probability threshold was set to 0.4. Following the removal of potential “contaminant ASVs” identified via the *decontam* *package* [21], we further investigated the possibility of “contaminant ASVs” remaining in the dataset. The latter was performed by generating a heatmap of relative abundances of the top 120 ASVs remaining in NTCs, ordered according to run number. Finally, we removed any remaining “spurious ASV” defined as ASVs with <10 reads across all biological specimens remaining in the dataset [6].

**Supplementary results**

**Table S1**. Characteristics of DCHS study population included in the LRTI case control analysis.

| Participant-level data |  |  |
| --- | --- | --- |
|  | qPCR data | 16S rRNA gene amplicon data^ϕ^ |
|  | N=544 | N=479 |
|  | n (%) | n (%) |
| Maternal smoking (self-report): |  |  |
| Yes | 151 (27.3) | 127 (26.5) |
| Maternal education: |  |  |
| Primary level | 52 (9.4) | 43 (9.0) |
| Started secondary level | 319 (57.6) | 277 (57.8) |
| Completed secondary level | 151 (27.3) | 132 (27.6) |
| Any tertiary level | 32 (5.8) | 27 (5.6) |
| Parent employed: |  |  |
| Yes | 275 (49.6) | 237 (49.5) |
| Household income (per month): |  |  |
| <1,000 ZAR | 187 (33.8) | 160 (33.4) |
| 1,000-5,000 ZAR | 302 (54.5) | 264 (55.1) |
| >5,000 ZAR | 65 (11.7) | 55 (11.5) |
| Household density, median (IQR) | 4 (3 - 6) | 4 (3 - 6) |
| Mode of delivery: |  |  |
| Vaginal delivery | 442 (80.1)^2^ | 382 (79.9)^1^ |
| Season of birth: |  |  |
| Summer | 152 (27.4) | 129 (26.9) |
| Autumn | 152 (27.4) | 136 (28.4) |
| Winter | 134 (24.2) | 114 (23.8) |
| Spring | 116 (20.9) | 100 (20.9) |
| WAZ at birth, median (IQR) | -0.3 (-0.0 - 0.4) | -0.2 (-0.9 - 0.4) |
| Gestational age: |  |  |
| Premature (<37 weeks gestation) | 93 (16.8) | 72 (15.0) |
| Sex: |  |  |
| Male | 294 (53.1) | 252 (52.6) |
| HIV^iv^-exposure: |  |  |
| HIV-exposed, uninfected | 148 (26.7) | 127 (26.5) |
| Exclusive breastfeeding (months), median (IQR) | 1.4 (0.5 - 3.0)^10^ | 1.1 (0.5-2.8) |
| Specimen-level data |  |  |
|  | qPCR data | 16S rRNA gene amplicon data^ϕ^ |
|  | N=888 | N=646 |
|  | n (%) | n (%) |
| Age at specimen collection (days), median (IQR) | 139 (81 - 220) | 128 (75 - 211) |
| Antibiotic use at specimen collection^φ^ |  |  |
| No | 799 (90.0) | 581 (89.9) |
| Antibiotic use commenced <24 hours prior to specimen collection | 55 (6.2) | 41 (6.4) |
| Antibiotic use commenced >24 hours and <7 days prior to specimen collection | 30 (3.4) | 24 (3.7) |
| Unknown | 4 (0.4) | 0 (0.0) |

*Superscript values represent missing values (n) in the dataset*

*^ϕ^Subset of qPCR data*

*^φ^No antibiotic use was reported for controls*

*LRTI, Lower respiratory tract infection; IQR, interquartile range; ZAR, South African Rand; WAZ: Weight-for-age z-score; HIV: Human immunodeficiency virus*

**Table S2**. Prevalence of viruses in specimens collected prior to LRTI diagnosis vs at or after LRTI diagnosis.

|  | Overall | Specimens collected prior to LRTI diagnosis | Specimens collected at or after LRTI diagnosis |  |
| --- | --- | --- | --- | --- |
|  |  | n (%) | n (%) |  |
|  | 323 | 13 (4.0) | 310 (96.0) | p-value |
| RSV (A/B) | 68 (21.1) | 2 (15.4) | 66 (21.3) | 0.609 |
| Adenovirus | 33 (10.2) | 1 (7.7) | 32 (10.3) | 0.759 |
| Rhinovirus | 103 (31.9) | 7 (53.9) | 96 (31.0) | 0.083 |
| Bocavirus | 12 (3.7) | 0 (0.0) | 12 (3.9) | 0.470 |
| Coronavirus | 29 (9.0) | 0 (0.0) | 29 (9.4) | 0.248 |
| Enterovirus | 34 (10.5) | 1 (7.7) | 33 (10.7) | 0.734 |
| Metapneumovirus (A/B) | 26 (8.1) | 0 (0.0) | 26 (8.4) | 0.276 |
| Influenza virus | 15 (4.6) | 0 (0.0) | 15 (4.8) | 0.417 |
| Cytomegalovirus | 133 (41.2) | 7 (53.9) | 126 (40.7) | 0.343 |
| Parainfluenza virus | 30 (9.3) | 2 (15.4) | 28 (9.0) | 0.440 |

*LRTI, Lower respiratory tract infection; RSV, Respiratory syncytial virus*

**Table S3**. Summary of clinical symptoms in children whose specimens were collected prior to LRTI diagnosis compared with specimens collected at or after LRTI diagnosis.

|  | Overall | Specimens collected prior to LRTI diagnosis | Specimens collected at or after LRTI diagnosis |  |
| --- | --- | --- | --- | --- |
|  | n (%) | n (%) | n (%) |  |
|  | 317* | 13 (4.0) | 310 (96.0) | p-value |
| Fever | 197/317 (62.2) | 5 (38.5) | 192 (63.2) | 0.072 |
| Cough | 306/317 (96.5) | 13 (100.0) | 293 (96.4) | 0.485 |
| Wheeze | 126 (39.8) | 6 (46.2) | 120 (39.5) | 0.630 |
| Vomiting | 37/316 (11.7) | 2 (15.4) | 35 (11.6) | 0.674 |
| Diarrhoea | 38/317 (12.0) | 0 (0.0) | 38 (12.5) | 0.174 |
| Blocked nose | 159/317 (50.2) | 4 (30.8) | 155 (60.0) | 0.153 |
| Runny nose | 170/317 (53.6) | 8 (61.5) | 162 (53.3) | 0.559 |

**Symptoms were not recorded for 6 case episodes*

*LRTI, Lower respiratory tract infection*

**Table S4**. Prevalence of viral and bacterial targets detected by qPCR in specimens collected at time of event, stratified by hospitalized or ambulatory LRTI

|  | Overall | No LRTI | Ambulatory LRTI | Hospitalised LRTI |  |
| --- | --- | --- | --- | --- | --- |
|  |  | n (%) | n (%) | n (%) |  |
|  | 646 | 323 (50.0) | 237 (36.7) | 86 (13.3) | p-value |
| **Bacteria** |  |  |  |  |  |
| *M. catarrhalis* | 467 (72.3) | 229 (70.9) | 182 (76.8) | 56 (65.1) | 0.085 |
| *H. influenzae* | 265 (41.0) | 106 (32.8) | 119 (50.2) | 40 (46.5) | <0.001 |
| *K. pneumoniae* | 78 (12.1) | 29 (9.0) | 37 (15.6) | 12 (14.0) | 0.050 |
| *S. pneumoniae* | 356 (55.1) | 179 (55.4) | 135 (57.0) | 42 (48.9) | 0.426 |
| *B. pertussis* | 7 (1.1) | 1 (0.3) | 4 (1.7) | 2 (2.3) | 0.146 |
| *S. aureus* | 145 (22.5) | 72 (22.3) | 54 (22.8) | 19 (22.1) | 0.987 |
| *M. pneumoniae* | 4 (0.6) | 3 (0.9) | 1 (0.4) | 0 (0.0) | 0.552 |
| *H. influenzae type B* | 10 (1.6) | 3 (0.9) | 6 (2.5) | 1 (1.2) | 0.301 |
| **Viruses** |  |  |  |  |  |
| RSV (A/B) | 84 (13.0) | 16 (5.0) | 37 (15.6) | 31 (36.1) | <0.001 |
| Rhinovirus | 189 (29.3) | 86 (26.6) | 82 (34.6) | 21 (24.4) | 0.070 |
| Parainfluenza virus | 40 (6.2) | 10 (3.1) | 23 (9.7) | 7 (8.1) | 0.004 |
| Adenovirus | 55 (8.5) | 22 (6.8) | 25 (10.6) | 8 (9.3) | 0.282 |
| Enterovirus | 48 (7.4) | 14 (4.3) | 31 (13.1) | 3 (3.5) | <0.001 |
| Coronavirus | 54 (8.4) | 25 (7.7) | 24 (10.1) | 5 (5.8) | 0.395 |
| Influenza virus | 18 (2.8) | 3 (0.9) | 12 (5.1) | 3 (3.5) | 0.012 |
| Metapneumovirus (A/B) | 45 (7.0) | 19 (5.9) | 23 (9.7) | 3 (3.5) | 0.085 |
| Cytomegalovirus | 263 (40.7) | 130 (40.3) | 98 (41.4) | 35 (40.7) | 0.966 |
| Bocavirus | 25 (3.9) | 13 (4.0) | 10 (4.2) | 2 (2.3) | 0.723 |

*LRTI, Lower respiratory tract infection; RSV, Respiratory syncytial virus*

**Molecular detection of nasopharyngeal pathogens commonly associated with LRTI**

**
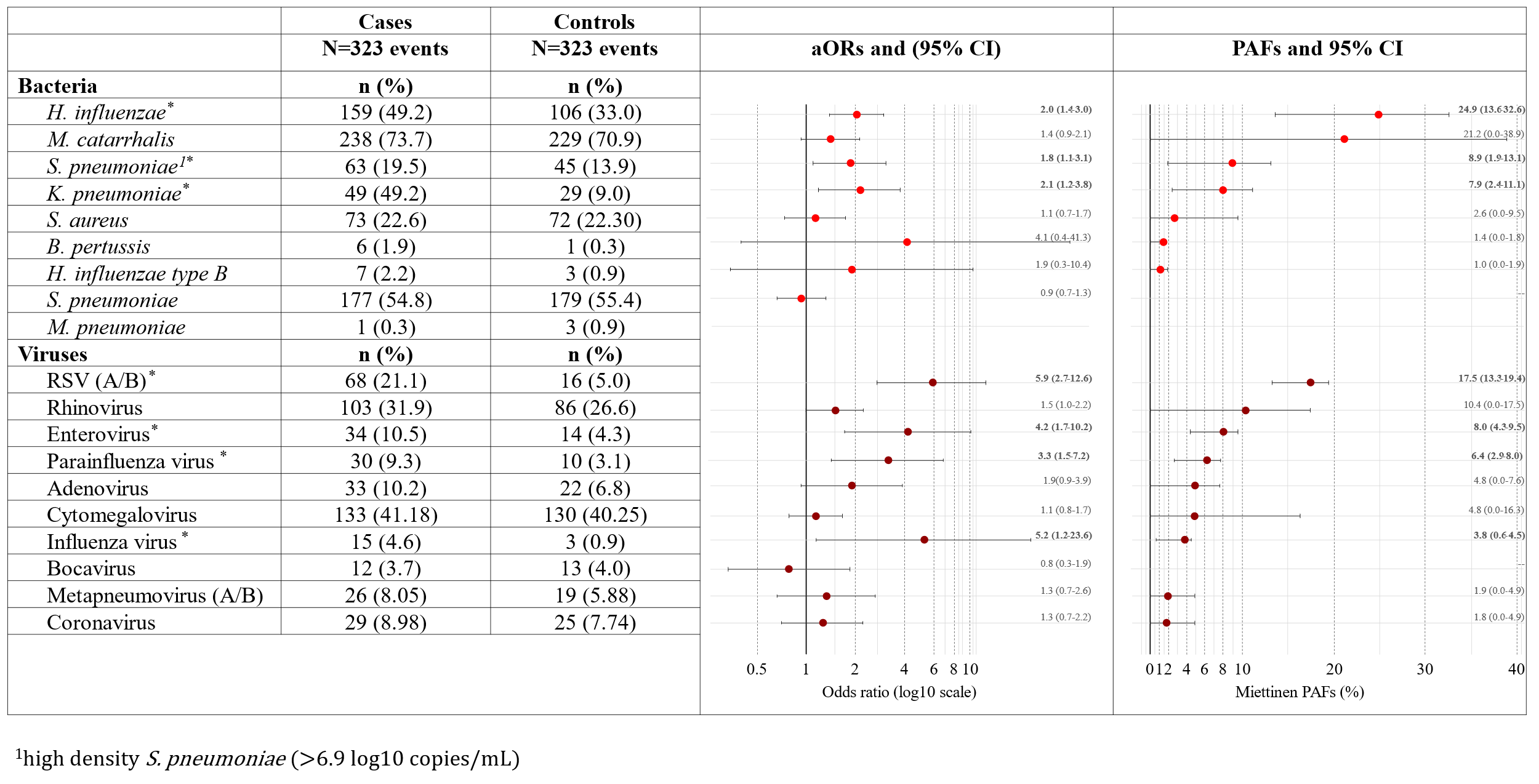
**

**Figure S3** **Associations between bacteria and viruses detected from nasopharyngeal (NP) specimens and lower respiratory tract infection (LRTI) during the first year of life.**

Adjusted odds ratios (aORs) and population attributable fractions (PAFs) calculated for each of the NP pathogens screened using the Fast-Track Diagnostics Respiratory Pathogens 33 (FTDResp33) test for a subgroup of participants (323 LRTI and 323 non-LRTI specimens) with 16S ribosomal ribonucleic acid (rRNA) gene amplicon data.

Significant associations are denoted by an asterisk.

**
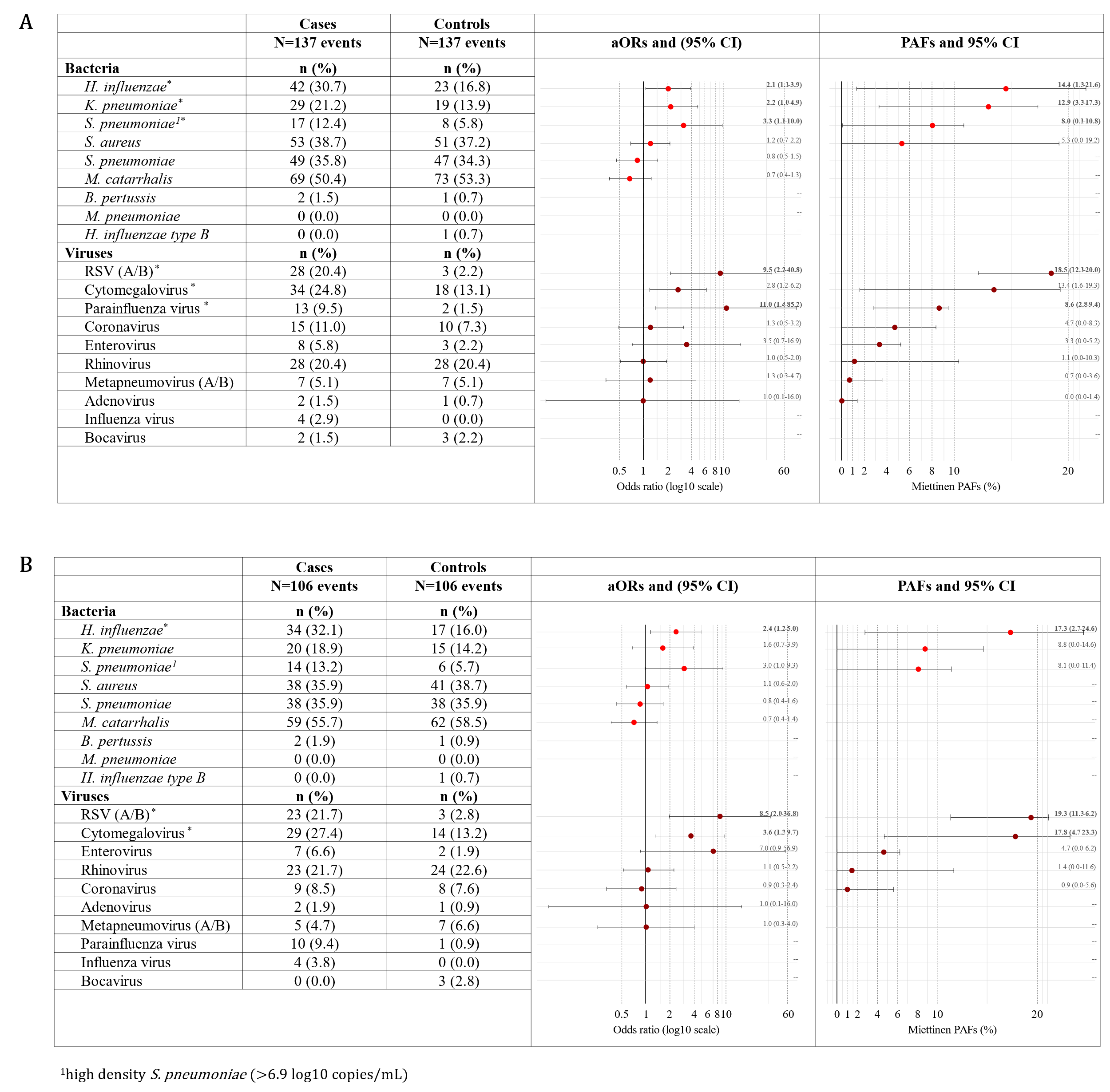
**

**Figure S4** **Associations between bacteria and viruses detected from nasopharyngeal (NP) specimens and lower respiratory tract infection (LRTI) during 0-3 months of life.**

Adjusted odds ratios (aORs) and population attributable fractions (PAFs) calculated for each of the NP pathogens screened using the Fast-Track Diagnostics Respiratory Pathogens 33 (FTDResp33) test for A) 137 LRTI and 137 non-LRTI specimens and B) a subgroup of 106 LRTI and 106 non-LRTI specimens with 16S ribosomal ribonucleic acid (rRNA) gene amplicon data. Significant associations are denoted by an asterisk.

**
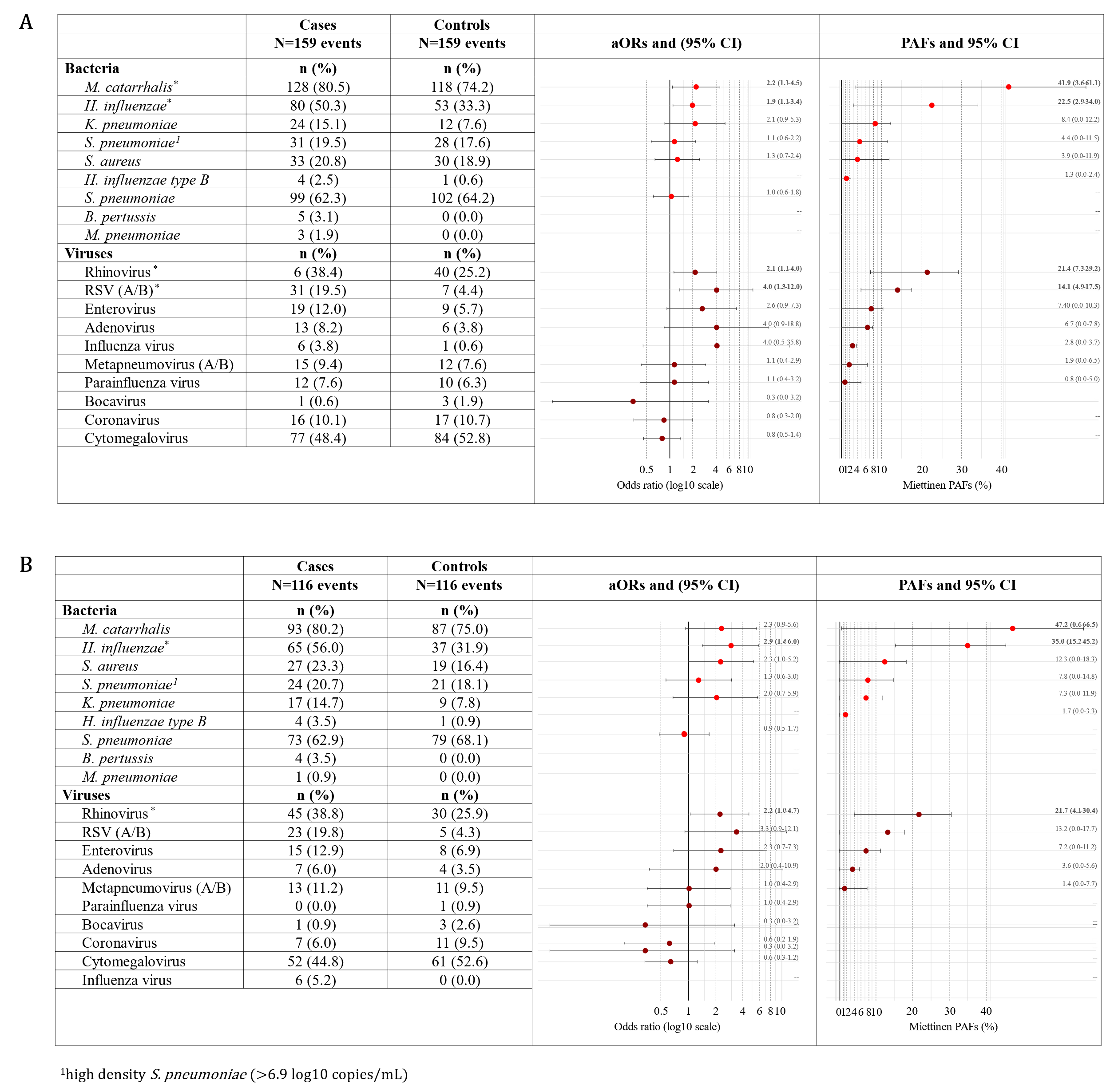
**

**Figure S5** **Associations between bacteria and viruses detected from nasopharyngeal (NP) specimens and lower respiratory tract infection (LRTI) during >3-6 months of life.**

Adjusted odds ratios (aORs) and population attributable fractions (PAFs) calculated for each of the NP pathogens screened using the Fast-Track Diagnostics Respiratory Pathogens 33 (FTDResp33) test for A) 159 LRTI and 159 non-LRTI specimens and B) a subgroup of 116 LRTI and 116 non-LRTI specimens with 16S ribosomal ribonucleic acid (rRNA) gene amplicon data. Significant associations are denoted by an asterisk.

**
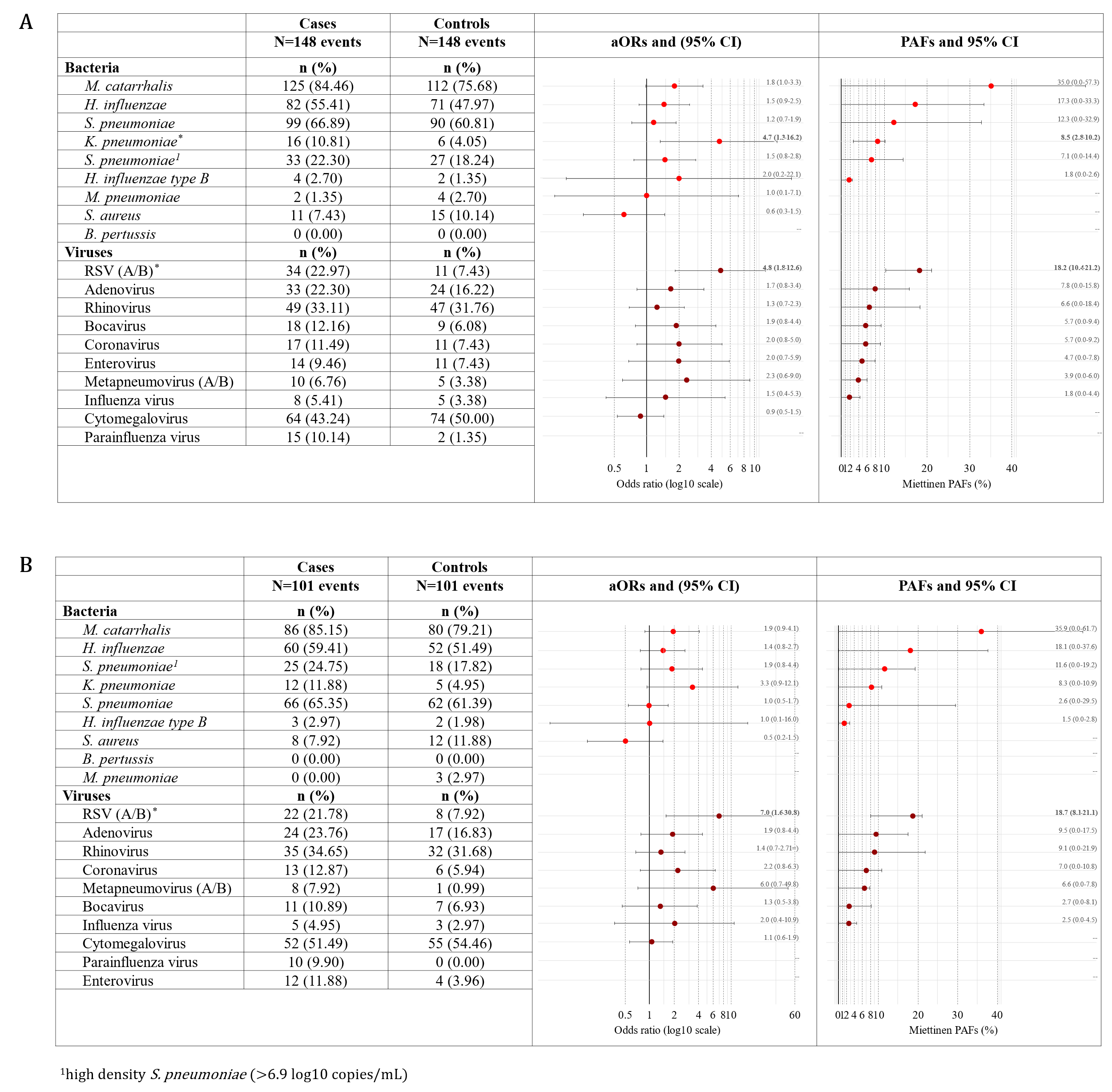
**

**Figure S6** **Associations between bacteria and viruses detected from nasopharyngeal (NP) specimens and lower respiratory tract infection (LRTI) during >6-12 months of life.**

Adjusted odds ratios (aORs) and population attributable fractions (PAFs) calculated for each of the NP pathogens screened using the Fast-Track Diagnostics Respiratory Pathogens 33 (FTDResp33) test for A) 148 LRTI and 148 non-LRTI specimens and B) a subgroup of 101 LRTI and 101 non-LRTI specimens with 16S ribosomal ribonucleic acid (rRNA) gene amplicon data. Significant associations are denoted by an asterisk.

**Table S5**. Prevalence of viruses in children with a single LRTI compared to children with ≥ 1 LRTI

|  | Overall | Single LRTI episode | ≥2 LRTI episodes |  |
| --- | --- | --- | --- | --- |
|  |  | n (%) | n (%) |  |
|  | 242 | 185 (76.5) | 57 (23.5) | P-value |
| RSV (A/B) | 63 (26.0) | 52 (28.1) | 11 (19.3) | 0.185 |
| Adenovirus | 22 (9.1) | 20 (10.8) | 2 (3.5) | 0.094 |
| Rhinovirus | 69 (28.5) | 49 (26.5) | 20 (35.1) | 0.209 |
| Bocavirus | 9 (3.7) | 9 (4.9) | 0.0 (0.0) | 0.090 |
| Coronavirus | 24 (9.9) | 19 (10.3) | 5 (8.8) | 0.741 |
| Enterovirus | 25 (10.3) | 21 (11.4) | 4 (7.0) | 0.347 |
| Metapneumovirus (A/B) | 18 (7.4) | 16 (8.7) | 2 (3.5) | 0.196 |
| Influenza virus | 9 (3.7) | 7 (3.8) | 2 (3.5) | 0.924 |
| Cytomegalovirus | 100 (41.3) | 80 (43.2) | 20 (35.1) | 0.274 |
| Parainfluenza virus | 23 (9.5) | 16 (8.7) | 7 (12.3) | 0.666 |

*LRTI, Lower respiratory tract infection; RSV, Respiratory syncytial virus*

**In-silico quality control of short read 16S ribosomal ribonucleic acid (rRNA) amplicon sequencing data**

*Sequencing reproducibility*

Bacterial profiles from 95 bacterial mock community controls [“BEI-DNA” (n=19) and “Zymobiomics-DNA” (n=76)] included in the 20 sequencing runs were reproducible and comparable to the theoretical compositions provided by the manufacturer (Figure S7 A-B).


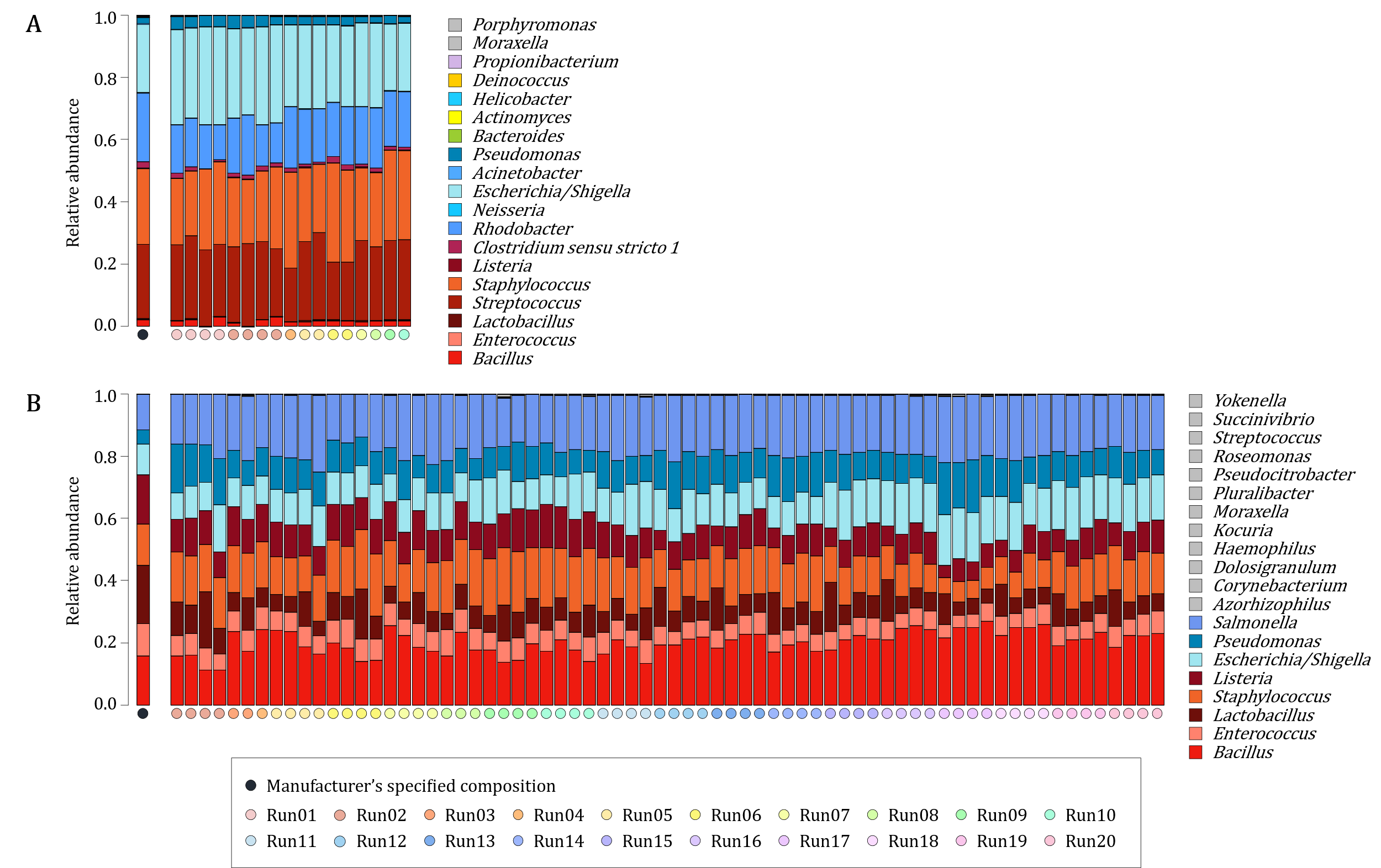


**Figure S7 Bacterial profiles sequenced from two sets of bacterial mock community DNA controls**

A) 1-in-10 fold dilutions of HM-783D (BEI Resources, NIAID, NIH as part of the Human Microbiome Project, VA, USA) (“BEI-DNA”, n=19), and B) 1-in-10 fold dilutions of ZymoBIOMICS™ Microbial Community DNA Standard (catalogue no. D6305, Zymo Research Corp., Irvine, CA, United States) (“Zymobiomics-DNA”, n=76)]. Manufacturers’ specified compositions for each of the bacterial mock community DNA controls are represented by navy circles at the bottom of each of the barplots. Rainbow-coloured circles at the bottom of each of the barplots represent different sequencing runs in which each of the profiles were generated. Taxonomy is assigned at genus-level, with colour-codes representing phylum-level classification (Shades of blue: Proteobacteria, shades of red: Firmicutes). Amplicon sequence variants (ASVs) detected from bacterial mock community DNA profiles which are not assigned to genera as per manufacturers’ specifications are shown in grey.

NP specimens randomly selected for repeat processing yielded high sequencing reproducibility across the 20 runs: “within-run” repeats processed in duplicate (n=72) and triplicate (n=2) [median R2 = 0.998 (IQR: 0.988–0.999)] and “between-run repeats” processed in duplicate (n=226), triplicate (n=26) and quadruplicate (n=17) [median R2 = 0.987 (IQR: 0.951–0.998)]. NP specimens collected at <10 days of age, NP specimens with <300 16S rRNA gene copies/μl and NP specimens with <1,000 reads were less reproducible compared to NP specimens collected at >10 days of age, NP specimens with >300 16S rRNA gene copies/μl and NP specimens with >1,000 reads, and resultantly flagged for exclusion (Figure S8 A-C).


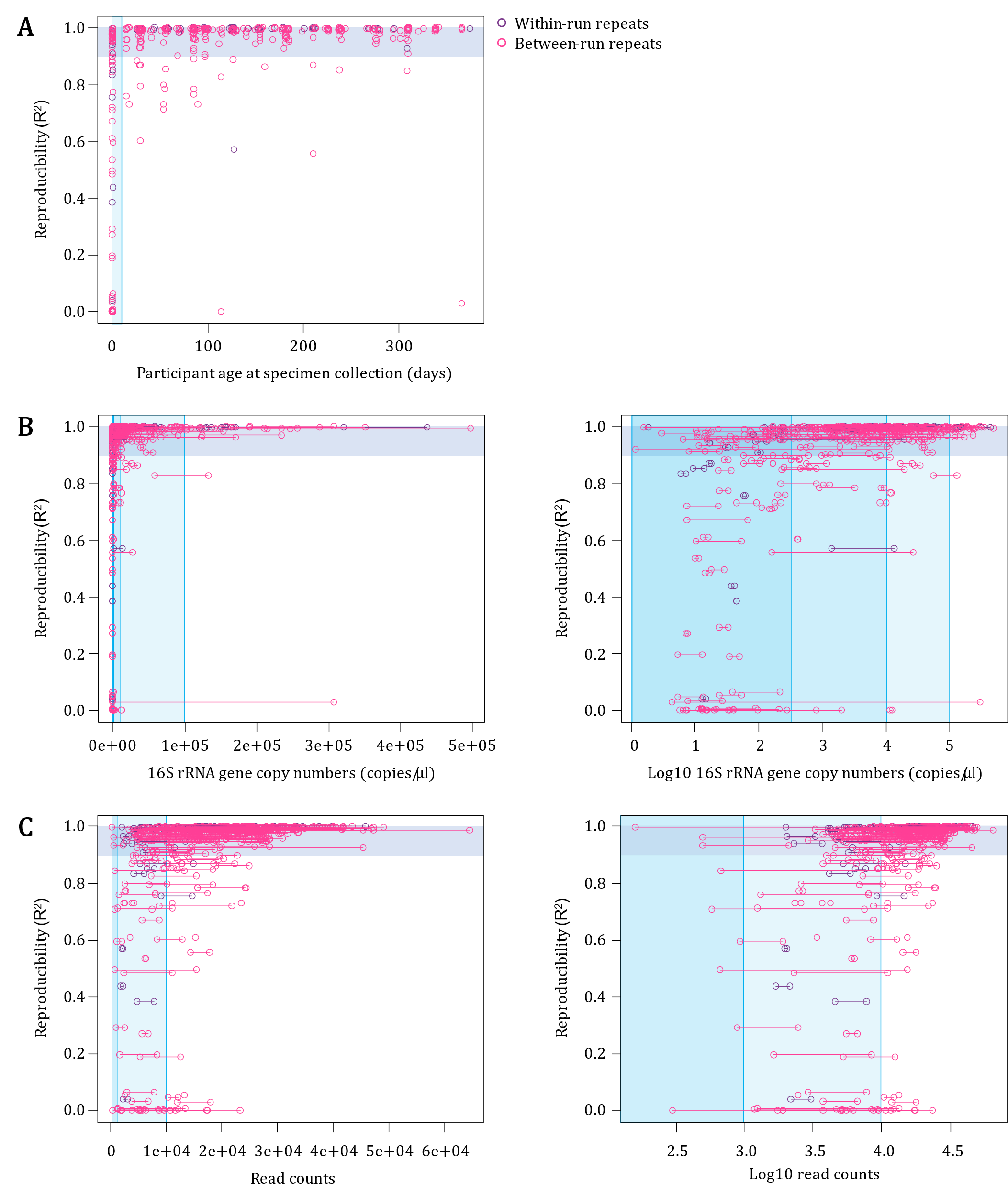


**Figure S8 Associations between reproducibility and A) participant age at specimen collection, B) 16S rRNA gene copy numbers, and C) read counts.**

Reproducibility is measured by coefficient of determination (R^2^) values, calculated by comparing proportions of each amplicon sequence variant (ASV) present between within-run or between-run repeats (technical repeats). Horizontal blue bars denote R^2^ values >0.90. Shades of vertical blue bars represent A) <10 days of age at the time of specimen collection; B) <300, <10,000, <100,000 16S rRNA gene copies/μl; and C) <1,000 and <10,000 reads; respectively. For B) and C), each set of within-run and between-run repeats had two 16S rRNA gene copy number/read count measures shown as two points connected by a horizontal line on the X-axis. Log10 values for B) and C) are shown on the right.

More NP specimens with <300 16S rRNA gene copies/μl (low biomass) had higher alpha diversity compared to NP specimens with >300 16S rRNA gene copies/μl (high biomass) (r=-0.141) (Figure S9 A). Median alpha diversity indices from NTCs and low biomass NP specimens were higher compared to median alpha diversity indices from high biomass NP specimens (Figure S9 B). Principal coordinate analysis (PCoA) plots of beta diversity showed that low biomass NP specimens clustered with NTCs[6] (Figure S9 C).


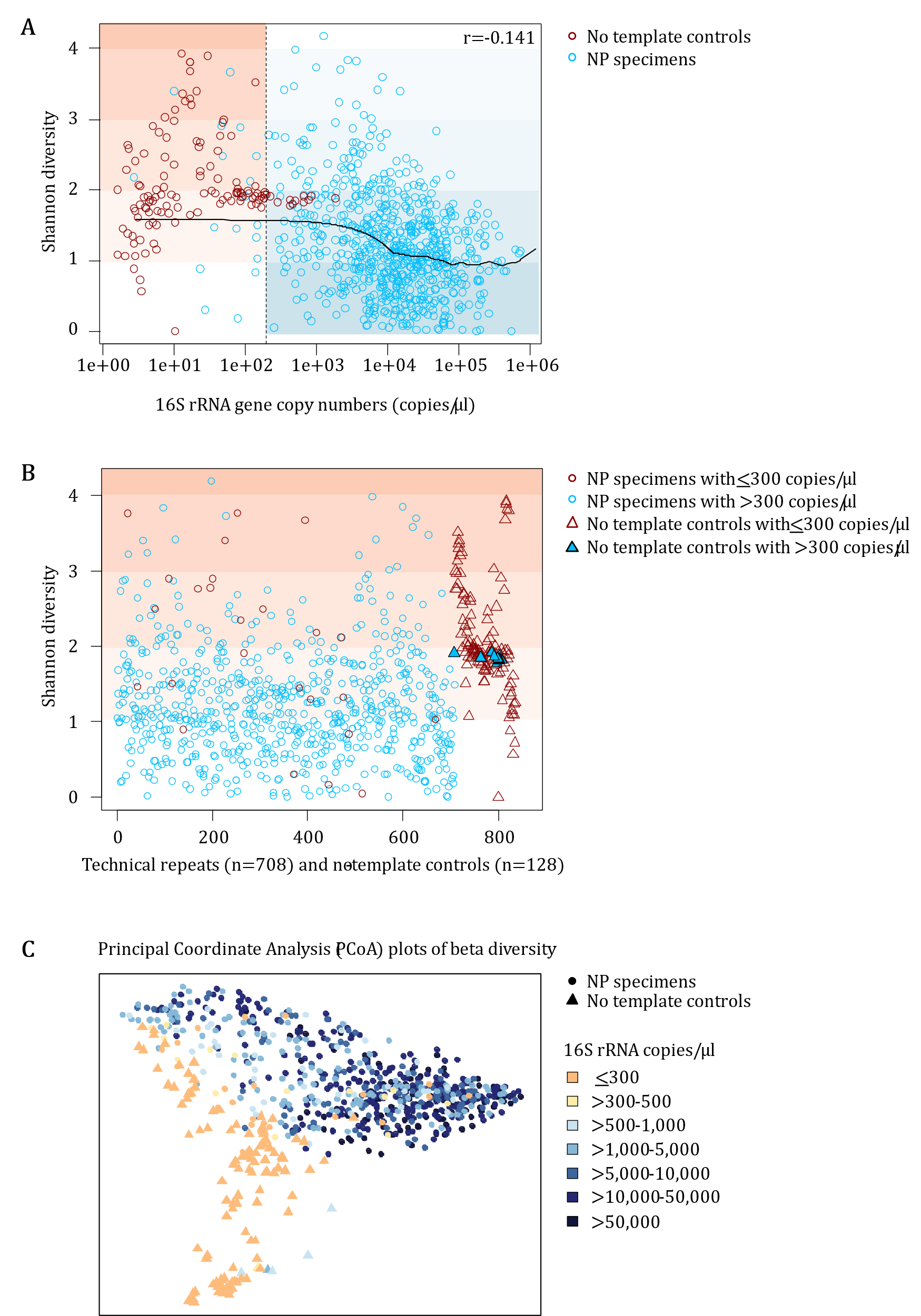


**Figure S9** **Diversity relative to specimen biomass for no template controls (NTCs) (n = 128) and nasopharyngeal (NP) specimens (n = 708)**

A) Scatter plot of alpha diversity (Shannon diversity) in relation to specimen biomass (16S rRNA gene copies/μl) for NTCs and NP specimens. Vertical orange and blue shaded areas highlight <300 and >300 16S rRNA gene copies/μl, respectively. B) Scatter plot of alpha diversity (Shannon diversity) for NTCs and NP specimens. Specimen biomass categories are denoted using red (<300 16S rRNA gene copies/μl) and blue (>300 16S rRNA gene copies/μl). C) Principal of coordinate analysis (PCoA) plots representing Bray-Curtis dissimilarities between specimens based on rarefied amplicon sequence variant (ASV) counts. Data points are coloured according to 16SrRNA gene copies/μl. Nasopharyngeal (NP) specimens are presented by filled circles. NTCs are presented by filled triangles.

A total of 708 NP specimens from 323 LRTI case-control sets were available for downstream analyses (Figure S10). After excluding NP specimens collected at <10 days of age (n=4), remaining NP specimens with <300 16S rRNA gene copies/µl (n=22), and remaining specimens with <1,000 reads (n=1), a total of 681 NP specimens were available for downstream analyses.


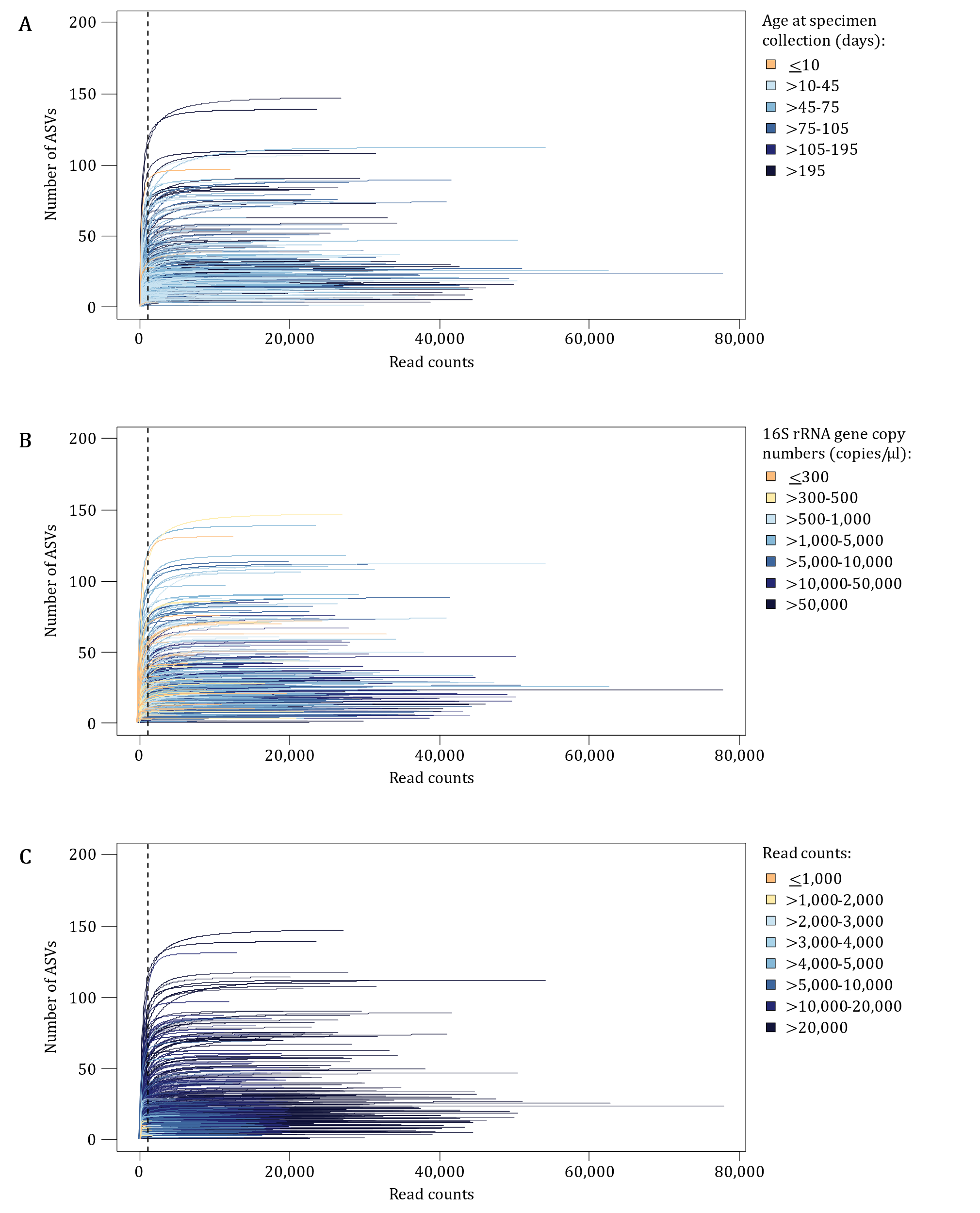


**Figure S10 Rarefaction curves prior to the removal of early life and low biomass nasopharyngeal (NP) specimens**

Rarefaction curves represent the number of amplicon sequence variants (ASVs) detected in relation to the read counts obtained from each NP specimen. The dotted vertical line denotes 1,000 reads. Rarefaction curves are coloured according to A) age at specimen collection, B) specimen biomass (16S rRNA gene copies/μl) and C) read counts.

*Identification and removal of potential contaminant amplicon sequence variants (ASVs)*

Additional exclusion of NP specimens matched to NP specimens collected at <10 days of age, NP specimens with <300 16S rRNA gene copies/µl and NP specimens with <1,000 reads resulted in 2,383 ASVs remaining in the dataset (646 NP specimens and 128 NTCs). A total of 138 “contaminant ASVs” (Table S5) were identified via the *decontam* package [6,21]. Exclusion of the 138 “contaminant ASVs” and ASVs unique to NTCs resulted in 1,473 ASVs remaining in the dataset (646 NP specimens and 128 NTCs). A heatmap of relative abundances of the top 120 ASVs in NTCs (n=128) revealed an additional 40 “contaminant ASVs” for exclusion (Table S5; Figure S11). A total of 605 “spurious ASVs” were further identified and removed resulting in 826 ASVs available for downstream analyses.

**Table S6**. “Contaminant ASVs” identified via the *decontam* package (n=138) and further investigation of NTC profiles (n=40)

|  | Phylum | Class | Order | Family | Genus | Method |
| --- | --- | --- | --- | --- | --- | --- |
| ASV_219 | Actinobacteriota | Actinobacteria | Bifidobacteriales | Bifidobacteriaceae | *Gardnerella* | decontam package |
| ASV_417 | Actinobacteriota | Actinobacteria | Corynebacteriales | Corynebacteriaceae | *Corynebacterium* | decontam package |
| ASV_397 | Actinobacteriota | Actinobacteria | Corynebacteriales | Corynebacteriaceae | *Corynebacterium* | decontam package |
| ASV_516 | Actinobacteriota | Actinobacteria | Corynebacteriales | Corynebacteriaceae | *Corynebacterium* | decontam package |
| ASV_359 | Actinobacteriota | Actinobacteria | Corynebacteriales | Corynebacteriaceae | *Lawsonella* | decontam package |
| ASV_11409 | Actinobacteriota | Actinobacteria | Corynebacteriales | Nocardiaceae | *Gordonia* | decontam package |
| ASV_218 | Actinobacteriota | Actinobacteria | Corynebacteriales | Nocardiaceae | *Rhodococcus* | NTC profiles |
| ASV_184 | Actinobacteriota | Actinobacteria | Frankiales | Geodermatophilaceae | *Blastococcus* | NTC profiles |
| ASV_2865 | Actinobacteriota | Actinobacteria | Frankiales | Geodermatophilaceae | *Geodermatophilus* | decontam package |
| ASV_848 | Actinobacteriota | Actinobacteria | Micrococcales | Brevibacteriaceae | *Brevibacterium* | decontam package |
| ASV_100 | Actinobacteriota | Actinobacteria | Micrococcales | Microbacteriaceae | *Yonghaparkia* | decontam package |
| ASV_540 | Actinobacteriota | Actinobacteria | Micrococcales | Micrococcaceae | *Kocuria* | decontam package |
| ASV_128 | Actinobacteriota | Actinobacteria | Micrococcales | Micrococcaceae | *Micrococcus* | decontam package |
| ASV_494 | Actinobacteriota | Actinobacteria | Micrococcales | Micrococcaceae | *Rothia* | decontam package |
| ASV_191 | Actinobacteriota | Actinobacteria | Propionibacteriales | Nocardioidaceae | *Nocardioides* | NTC profiles |
| ASV_553 | Actinobacteriota | Actinobacteria | Propionibacteriales | Nocardioidaceae | *Nocardioides* | decontam package |
| ASV_309 | Actinobacteriota | Actinobacteria | Propionibacteriales | Nocardioidaceae | *Nocardioides* | decontam package |
| ASV_266 | Actinobacteriota | Actinobacteria | Propionibacteriales | Propionibacteriaceae | *Cutibacterium* | NTC profiles |
| ASV_1630 | Bacteroidota | Bacteroidia | Bacteroidales | Porphyromonadaceae | *Porphyromonas* | decontam package |
| ASV_342 | Bacteroidota | Bacteroidia | Bacteroidales | Prevotellaceae | *Prevotella* | decontam package |
| ASV_2422 | Bacteroidota | Bacteroidia | Bacteroidales | Prevotellaceae | *Prevotella* | decontam package |
| ASV_58495 | Bacteroidota | Bacteroidia | Chitinophagales | Chitinophagaceae | *Aurantisolimonas* | decontam package |

*ASV. Amplicon sequence variant; NTC, No template control; A-N-P-R*: *Allorhizobium-Neorhizobium-Pararhizobium-Rhizobium*

**Table S6**. “Contaminant ASVs” identified via the *decontam* package (n=138) and further investigation of NTC profiles (n=40) (continued)

|  | Phylum | Class | Order | Family | Genus | Method |
| --- | --- | --- | --- | --- | --- | --- |
| ASV_2765 | Bacteroidota | Bacteroidia | Chitinophagales | Chitinophagaceae | *Cnuella* | decontam package |
| ASV_279 | Bacteroidota | Bacteroidia | Chitinophagales | Chitinophagaceae | *Cnuella* | decontam package |
| ASV_535 | Bacteroidota | Bacteroidia | Chitinophagales | Chitinophagaceae | *Flavisolibacter* | decontam package |
| ASV_17387 | Bacteroidota | Bacteroidia | Cytophagales | Hymenobacteraceae | *Pontibacter* | decontam package |
| ASV_548 | Bacteroidota | Bacteroidia | Flavobacteriales | Weeksellaceae | *Cloacibacterium* | decontam package |
| ASV_15763 | Bdellovibrionota | Bdellovibrionia | Bacteriovoracales | Bacteriovoracaceae | *Peredibacter* | decontam package |
| ASV_578 | Bdellovibrionota | Bdellovibrionia | Bacteriovoracales | Bacteriovoracaceae | *Peredibacter* | decontam package |
| ASV_143 | Campilobacterota | Campylobacteria | Campylobacterales | Helicobacteraceae | unclassified | decontam package |
| ASV_545 | Cyanobacteria | Cyanobacteriia | Chloroplast | unclassified | unclassified | decontam package |
| ASV_254 | Cyanobacteria | Cyanobacteriia | Chloroplast | unclassified | unclassified | decontam package |
| ASV_663 | Cyanobacteria | Cyanobacteriia | Chloroplast | unclassified | unclassified | decontam package |
| ASV_8271 | Cyanobacteria | Sericytochromatia | unclassified | unclassified | unclassified | decontam package |
| ASV_698 | Deinococcota | Deinococci | Deinococcales | Deinococcaceae | *Deinococcus* | decontam package |
| ASV_723 | Deinococcota | Deinococci | Deinococcales | Deinococcaceae | *Deinococcus* | decontam package |
| ASV_716 | Firmicutes | Bacilli | Bacillales | Planococcaceae | *Planomicrobium* | decontam package |
| ASV_790 | Firmicutes | Bacilli | Lactobacillales | Aerococcaceae | *Aerococcus* | decontam package |
| ASV_92 | Firmicutes | Bacilli | Lactobacillales | Enterococcaceae | *Enterococcus* | decontam package |
| ASV_47 | Firmicutes | Bacilli | Lactobacillales | Lactobacillaceae | *Lactobacillus* | NTC profiles |
| ASV_1372 | Firmicutes | Bacilli | Lactobacillales | Streptococcaceae | *Lactococcus* | decontam package |
| ASV_87 | Firmicutes | Bacilli | Lactobacillales | Streptococcaceae | *Streptococcus* | decontam package |
| ASV_674 | Firmicutes | Bacilli | Lactobacillales | Streptococcaceae | *Streptococcus* | decontam package |
| ASV_249 | Firmicutes | Bacilli | Mycoplasmatales | Mycoplasmataceae | *Mycoplasma* | decontam package |
| ASV_391 | Fusobacteriota | Fusobacteriia | Fusobacteriales | Fusobacteriaceae | *Fusobacterium* | decontam package |
| ASV_648 | Patescibacteria | Saccharimonadia | Saccharimonadales | LWQ8 | unclassified | decontam package |

*ASV. Amplicon sequence variant; NTC, No template control; A-N-P-R*: *Allorhizobium-Neorhizobium-Pararhizobium-Rhizobium*

**Table S6**. “Contaminant ASVs” identified via the *decontam* package (n=138) and further investigation of NTC profiles (n=40) (continued)

|  | Phylum | Class | Order | Family | Genus | Method |
| --- | --- | --- | --- | --- | --- | --- |
| ASV_366 | Proteobacteria | Alphaproteobacteria | Azospirillales | Azospirillaceae | *Azospirillum* | NTC profiles |
| ASV_83 | Proteobacteria | Alphaproteobacteria | Azospirillales | Azospirillaceae | *Azospirillum* | NTC profiles |
| ASV_137 | Proteobacteria | Alphaproteobacteria | Azospirillales | Azospirillaceae | unclassified | decontam package |
| ASV_157 | Proteobacteria | Alphaproteobacteria | Azospirillales | Azospirillaceae | unclassified | NTC profiles |
| ASV_48 | Proteobacteria | Alphaproteobacteria | Azospirillales | Azospirillaceae | unclassified | NTC profiles |
| ASV_623 | Proteobacteria | Alphaproteobacteria | Azospirillales | Azospirillaceae | *Nitrospirillum* | decontam package |
| ASV_215 | Proteobacteria | Alphaproteobacteria | Azospirillales | Azospirillaceae | *Rhodocista* | NTC profiles |
| ASV_326 | Proteobacteria | Alphaproteobacteria | Caulobacterales | Caulobacteraceae | *Brevundimonas* | decontam package |
| ASV_436 | Proteobacteria | Alphaproteobacteria | Caulobacterales | Caulobacteraceae | *Brevundimonas* | decontam package |
| ASV_378 | Proteobacteria | Alphaproteobacteria | Caulobacterales | Caulobacteraceae | *Brevundimonas* | decontam package |
| ASV_130 | Proteobacteria | Alphaproteobacteria | Caulobacterales | Caulobacteraceae | *Brevundimonas* | decontam package |
| ASV_1118 | Proteobacteria | Alphaproteobacteria | Caulobacterales | Caulobacteraceae | *Brevundimonas* | decontam package |
| ASV_133 | Proteobacteria | Alphaproteobacteria | Caulobacterales | Caulobacteraceae | *Brevundimonas* | decontam package |
| ASV_624 | Proteobacteria | Alphaproteobacteria | Caulobacterales | Caulobacteraceae | *Brevundimonas* | decontam package |
| ASV_239 | Proteobacteria | Alphaproteobacteria | Caulobacterales | Caulobacteraceae | *Brevundimonas* | NTC profiles |
| ASV_174 | Proteobacteria | Alphaproteobacteria | Caulobacterales | Caulobacteraceae | *Caulobacter* | decontam package |
| ASV_78 | Proteobacteria | Alphaproteobacteria | Caulobacterales | Caulobacteraceae | unclassified | decontam package |
| ASV_3196 | Proteobacteria | Alphaproteobacteria | Caulobacterales | Caulobacteraceae | *Phenylobacterium* | decontam package |
| ASV_513 | Proteobacteria | Alphaproteobacteria | Caulobacterales | Caulobacteraceae | *Phenylobacterium* | decontam package |
| ASV_182 | Proteobacteria | Alphaproteobacteria | Caulobacterales | Caulobacteraceae | *Phenylobacterium* | NTC profiles |
| ASV_456 | Proteobacteria | Alphaproteobacteria | Caulobacterales | Caulobacteraceae | *Phenylobacterium* | NTC profiles |
| ASV_193 | Proteobacteria | Alphaproteobacteria | Caulobacterales | Caulobacteraceae | *PMMR1* | decontam package |
| ASV_3425 | Proteobacteria | Alphaproteobacteria | Caulobacterales | Caulobacteraceae | *PMMR1* | decontam package |
| ASV_385 | Proteobacteria | Alphaproteobacteria | Caulobacterales | Caulobacteraceae | *PMMR1* | decontam package |

*ASV. Amplicon sequence variant; NTC, No template control; A-N-P-R*: *Allorhizobium-Neorhizobium-Pararhizobium-Rhizobium*

**Table S6**. “Contaminant ASVs” identified via the *decontam* package (n=138) and further investigation of NTC profiles (n=40) (continued)

|  | Phylum | Class | Order | Family | Genus | Method |
| --- | --- | --- | --- | --- | --- | --- |
| ASV_770 | Proteobacteria | Alphaproteobacteria | Caulobacterales | Caulobacteraceae | *PMMR1* | NTC profiles |
| ASV_91 | Proteobacteria | Alphaproteobacteria | Caulobacterales | Caulobacteraceae | *PMMR1* | NTC profiles |
| ASV_425 | Proteobacteria | Alphaproteobacteria | Micavibrionales | unclassified | unclassified | decontam package |
| ASV_929 | Proteobacteria | Alphaproteobacteria | unclassified | unclassified | unclassified | decontam package |
| ASV_58 | Proteobacteria | Alphaproteobacteria | Rhizobiales | Beijerinckiaceae | *Bosea* | decontam package |
| ASV_761 | Proteobacteria | Alphaproteobacteria | Rhizobiales | Beijerinckiaceae | *Chelatococcus* | decontam package |
| ASV_370 | Proteobacteria | Alphaproteobacteria | Rhizobiales | Beijerinckiaceae | *Methylobacterium-Methylorubrum* | decontam package |
| ASV_2279 | Proteobacteria | Alphaproteobacteria | Rhizobiales | Beijerinckiaceae | *Methylobacterium-Methylorubrum* | decontam package |
| ASV_357 | Proteobacteria | Alphaproteobacteria | Rhizobiales | Beijerinckiaceae | *Methylobacterium-Methylorubrum* | decontam package |
| ASV_547 | Proteobacteria | Alphaproteobacteria | Rhizobiales | Beijerinckiaceae | *Microvirga* | decontam package |
| ASV_323 | Proteobacteria | Alphaproteobacteria | Rhizobiales | Beijerinckiaceae | unclassified | decontam package |
| ASV_183 | Proteobacteria | Alphaproteobacteria | Rhizobiales | Beijerinckiaceae | *Salinarimonas* | NTC profiles |
| ASV_2053 | Proteobacteria | Alphaproteobacteria | Rhizobiales | Devosiaceae | *Devosia* | decontam package |
| ASV_364 | Proteobacteria | Alphaproteobacteria | Rhizobiales | Devosiaceae | *Devosia* | decontam package |
| ASV_171 | Proteobacteria | Alphaproteobacteria | Rhizobiales | Pleomorphomonadaceae | *Chthonobacter* | decontam package |
| ASV_1038 | Proteobacteria | Alphaproteobacteria | Rhizobiales | Pleomorphomonadaceae | *Chthonobacter* | decontam package |
| ASV_106 | Proteobacteria | Alphaproteobacteria | Rhizobiales | Rhizobiaceae | *ANPR* | decontam package |
| ASV_89 | Proteobacteria | Alphaproteobacteria | Rhizobiales | Rhizobiaceae | *ANPR* | NTC profiles |
| ASV_600 | Proteobacteria | Alphaproteobacteria | Rhizobiales | Rhizobiaceae | *Aureimonas* | decontam package |
| ASV_210 | Proteobacteria | Alphaproteobacteria | Rhizobiales | Rhizobiaceae | *Aureimonas* | NTC profiles |
| ASV_797 | Proteobacteria | Alphaproteobacteria | Rhizobiales | Rhizobiaceae | *Aureimonas* | NTC profiles |
| ASV_961 | Proteobacteria | Alphaproteobacteria | Rhizobiales | Rhizobiaceae | *Aureimonas* | NTC profiles |
| ASV_660 | Proteobacteria | Alphaproteobacteria | Rhizobiales | Rhizobiaceae | *Pseudorhizobium* | decontam package |
| ASV_167 | Proteobacteria | Alphaproteobacteria | Rhizobiales | Stappiaceae | *Pannonibacter* | decontam package |

*ASV. Amplicon sequence variant; NTC, No template control; A-N-P-R*: *Allorhizobium-Neorhizobium-Pararhizobium-Rhizobium*

**Table S6.** “Contaminant ASVs” identified via the *decontam* package (n=138) and further investigation of NTC profiles (n=40) (continued)

|  | Phylum | Class | Order | Family | Genus | Method |
| --- | --- | --- | --- | --- | --- | --- |
| ASV_1365 | Proteobacteria | Alphaproteobacteria | Rhizobiales | Xanthobacteraceae | *Azorhizobium* | NTC profiles |
| ASV_858 | Proteobacteria | Alphaproteobacteria | Rhodobacterales | Rhodobacteraceae | *Cereibacter* | decontam package |
| ASV_383 | Proteobacteria | Alphaproteobacteria | Rhodobacterales | Rhodobacteraceae | *Paracoccus* | decontam package |
| ASV_351 | Proteobacteria | Alphaproteobacteria | Rhodobacterales | Rhodobacteraceae | *Paracoccus* | decontam package |
| ASV_22 | Proteobacteria | Alphaproteobacteria | Rhodobacterales | Rhodobacteraceae | *Paracoccus* | NTC profiles |
| ASV_629 | Proteobacteria | Alphaproteobacteria | Rhodobacterales | Rhodobacteraceae | *Rubellimicrobium* | NTC profiles |
| ASV_409 | Proteobacteria | Alphaproteobacteria | Rhodospirillales | Rhodospirillaceae | *Novispirillum* | decontam package |
| ASV_23 | Proteobacteria | Alphaproteobacteria | Sphingomonadales | Sphingomonadaceae | *Blastomonas* | decontam package |
| ASV_1021 | Proteobacteria | Alphaproteobacteria | Sphingomonadales | Sphingomonadaceae | unclassified | decontam package |
| ASV_3882 | Proteobacteria | Alphaproteobacteria | Sphingomonadales | Sphingomonadaceae | *Novosphingobium* | NTC profiles |
| ASV_273 | Proteobacteria | Alphaproteobacteria | Sphingomonadales | Sphingomonadaceae | *Porphyrobacter* | NTC profiles |
| ASV_607 | Proteobacteria | Alphaproteobacteria | Sphingomonadales | Sphingomonadaceae | *Qipengyuania* | decontam package |
| ASV_1221 | Proteobacteria | Alphaproteobacteria | Sphingomonadales | Sphingomonadaceae | *Sphingomonas* | decontam package |
| ASV_739 | Proteobacteria | Alphaproteobacteria | Sphingomonadales | Sphingomonadaceae | *Sphingomonas* | decontam package |
| ASV_846 | Proteobacteria | Alphaproteobacteria | Sphingomonadales | Sphingomonadaceae | *Sphingomonas* | decontam package |
| ASV_1207 | Proteobacteria | Alphaproteobacteria | Sphingomonadales | Sphingomonadaceae | *Sphingomonas* | decontam package |
| ASV_178 | Proteobacteria | Alphaproteobacteria | Sphingomonadales | Sphingomonadaceae | *Sphingomonas* | NTC profiles |
| ASV_1017 | Proteobacteria | Gammaproteobacteria | Alteromonadales | Alteromonadaceae | *Rheinheimera* | decontam package |
| ASV_165 | Proteobacteria | Gammaproteobacteria | Alteromonadales | Alteromonadaceae | *Rheinheimera* | decontam package |
| ASV_138 | Proteobacteria | Gammaproteobacteria | Alteromonadales | Shewanellaceae | *Shewanella* | decontam package |
| ASV_363 | Proteobacteria | Gammaproteobacteria | Burkholderiales | Burkholderiaceae | *Cupriavidus* | decontam package |
| ASV_139 | Proteobacteria | Gammaproteobacteria | Burkholderiales | Chromobacteriaceae | *Vogesella* | NTC profiles |
| ASV_52 | Proteobacteria | Gammaproteobacteria | Burkholderiales | Comamonadaceae | *Acidovorax* | decontam package |
| ASV_1180 | Proteobacteria | Gammaproteobacteria | Burkholderiales | Comamonadaceae | *Aquabacterium* | decontam package |

*ASV. Amplicon sequence variant; NTC, No template control; A-N-P-R*: *Allorhizobium-Neorhizobium-Pararhizobium-Rhizobium*

**Table S6.** “Contaminant ASVs” identified via the *decontam* package (n=138) and further investigation of NTC profiles (n=40) (continued)

|  | Phylum | Class | Order | Family | Genus | Method |
| --- | --- | --- | --- | --- | --- | --- |
| ASV_82 | Proteobacteria | Gammaproteobacteria | Burkholderiales | Comamonadaceae | *Aquabacterium* | NTC profiles |
| ASV_1392 | Proteobacteria | Gammaproteobacteria | Burkholderiales | Comamonadaceae | *Caenimonas* | decontam package |
| ASV_719 | Proteobacteria | Gammaproteobacteria | Burkholderiales | Comamonadaceae | *Hydrogenophaga* | decontam package |
| ASV_246 | Proteobacteria | Gammaproteobacteria | Burkholderiales | Comamonadaceae | *Hydrogenophaga* | decontam package |
| ASV_1505 | Proteobacteria | Gammaproteobacteria | Burkholderiales | Comamonadaceae | *Hydrogenophaga* | decontam package |
| ASV_111 | Proteobacteria | Gammaproteobacteria | Burkholderiales | Comamonadaceae | unclassified | decontam package |
| ASV_299 | Proteobacteria | Gammaproteobacteria | Burkholderiales | Comamonadaceae | unclassified | decontam package |
| ASV_26 | Proteobacteria | Gammaproteobacteria | Burkholderiales | Comamonadaceae | unclassified | NTC profiles |
| ASV_442 | Proteobacteria | Gammaproteobacteria | Burkholderiales | Comamonadaceae | *Ottowia* | decontam package |
| ASV_121 | Proteobacteria | Gammaproteobacteria | Burkholderiales | Comamonadaceae | *Piscinibacter* | decontam package |
| ASV_190 | Proteobacteria | Gammaproteobacteria | Burkholderiales | Comamonadaceae | *Ramlibacter* | decontam package |
| ASV_784 | Proteobacteria | Gammaproteobacteria | Burkholderiales | Comamonadaceae | *Rhodoferax* | decontam package |
| ASV_90 | Proteobacteria | Gammaproteobacteria | Burkholderiales | Comamonadaceae | *Rubrivivax* | NTC profiles |
| ASV_520 | Proteobacteria | Gammaproteobacteria | Burkholderiales | Comamonadaceae | *Schlegelella* | decontam package |
| ASV_20038 | Proteobacteria | Gammaproteobacteria | Burkholderiales | unclassified | unclassified | decontam package |
| ASV_679 | Proteobacteria | Gammaproteobacteria | Burkholderiales | unclassified | unclassified | decontam package |
| ASV_159 | Proteobacteria | Gammaproteobacteria | Burkholderiales | Oxalobacteraceae | *Massilia* | decontam package |
| ASV_341 | Proteobacteria | Gammaproteobacteria | Burkholderiales | Oxalobacteraceae | *Massilia* | decontam package |
| ASV_117 | Proteobacteria | Gammaproteobacteria | Burkholderiales | Oxalobacteraceae | *Massilia* | NTC profiles |
| ASV_41 | Proteobacteria | Gammaproteobacteria | Burkholderiales | Oxalobacteraceae | *Massilia* | NTC profiles |
| ASV_24 | Proteobacteria | Gammaproteobacteria | Burkholderiales | Oxalobacteraceae | *Noviherbaspirillum* | NTC profiles |
| ASV_39 | Proteobacteria | Gammaproteobacteria | Burkholderiales | Oxalobacteraceae | *Noviherbaspirillum* | NTC profiles |
| ASV_50 | Proteobacteria | Gammaproteobacteria | Burkholderiales | Oxalobacteraceae | *Noviherbaspirillum* | NTC profiles |
| ASV_80 | Proteobacteria | Gammaproteobacteria | Burkholderiales | Oxalobacteraceae | *Noviherbaspirillum* | NTC profiles |

*ASV. Amplicon sequence variant; NTC, No template control; A-N-P-R*: *Allorhizobium-Neorhizobium-Pararhizobium-Rhizobium*

**Table S6.** “Contaminant ASVs” identified via the *decontam* package (n=138) and further investigation of NTC profiles (n=40) (continued)

|  | Phylum | Class | Order | Family | Genus | Method |
| --- | --- | --- | --- | --- | --- | --- |
| ASV_173 | Proteobacteria | Gammaproteobacteria | Burkholderiales | Oxalobacteraceae | *Paucimonas* | NTC profiles |
| ASV_12 | Proteobacteria | Gammaproteobacteria | Enterobacterales | Enterobacteriaceae | *Escherichia/Shigella* | decontam package |
| ASV_205 | Proteobacteria | Gammaproteobacteria | Enterobacterales | Enterobacteriaceae | *Klebsiella* | decontam package |
| ASV_145 | Proteobacteria | Gammaproteobacteria | Enterobacterales | Enterobacteriaceae | unclassified | decontam package |
| ASV_534 | Proteobacteria | Gammaproteobacteria | Pasteurellales | Pasteurellaceae | unclassified | decontam package |
| ASV_250 | Proteobacteria | Gammaproteobacteria | Pseudomonadales | Moraxellaceae | *Acinetobacter* | decontam package |
| ASV_95 | Proteobacteria | Gammaproteobacteria | Pseudomonadales | Moraxellaceae | *Acinetobacter* | decontam package |
| ASV_88 | Proteobacteria | Gammaproteobacteria | Pseudomonadales | Moraxellaceae | *Acinetobacter* | decontam package |
| ASV_85 | Proteobacteria | Gammaproteobacteria | Pseudomonadales | Moraxellaceae | *Acinetobacter* | decontam package |
| ASV_66 | Proteobacteria | Gammaproteobacteria | Pseudomonadales | Moraxellaceae | *Acinetobacter* | decontam package |
| ASV_252 | Proteobacteria | Gammaproteobacteria | Pseudomonadales | Moraxellaceae | *Acinetobacter* | decontam package |
| ASV_18873 | Proteobacteria | Gammaproteobacteria | Pseudomonadales | Moraxellaceae | *Acinetobacter* | decontam package |
| ASV_866 | Proteobacteria | Gammaproteobacteria | Pseudomonadales | Moraxellaceae | *Acinetobacter* | decontam package |
| ASV_291 | Proteobacteria | Gammaproteobacteria | Pseudomonadales | Moraxellaceae | *Alkanindiges* | NTC profiles |
| ASV_126 | Proteobacteria | Gammaproteobacteria | Pseudomonadales | Moraxellaceae | *Enhydrobacter* | NTC profiles |
| ASV_4595 | Proteobacteria | Gammaproteobacteria | Pseudomonadales | Moraxellaceae | *Moraxella* | decontam package |
| ASV_7596 | Proteobacteria | Gammaproteobacteria | Pseudomonadales | Moraxellaceae | *Moraxella* | decontam package |
| ASV_310 | Proteobacteria | Gammaproteobacteria | Pseudomonadales | Pseudomonadaceae | *Pseudomonas* | decontam package |
| ASV_142 | Proteobacteria | Gammaproteobacteria | Pseudomonadales | Pseudomonadaceae | *Pseudomonas* | decontam package |
| ASV_44 | Proteobacteria | Gammaproteobacteria | Pseudomonadales | Pseudomonadaceae | *Pseudomonas* | decontam package |
| ASV_313 | Proteobacteria | Gammaproteobacteria | Pseudomonadales | Pseudomonadaceae | *Pseudomonas* | decontam package |
| ASV_204 | Proteobacteria | Gammaproteobacteria | Pseudomonadales | Pseudomonadaceae | *Pseudomonas* | decontam package |
| ASV_168 | Proteobacteria | Gammaproteobacteria | Pseudomonadales | Pseudomonadaceae | *Pseudomonas* | decontam package |
| ASV_228 | Proteobacteria | Gammaproteobacteria | Pseudomonadales | Pseudomonadaceae | *Pseudomonas* | decontam package |

*ASV. Amplicon sequence variant; NTC, No template control; A-N-P-R*: *Allorhizobium-Neorhizobium-Pararhizobium-Rhizobium*

**Table S6.** “Contaminant ASVs” identified via the *decontam* package (n=138) and further investigation of NTC profiles (n=40) (continued)

|  | Phylum | Class | Order | Family | Genus | Method |
| --- | --- | --- | --- | --- | --- | --- |
| ASV_517 | Proteobacteria | Gammaproteobacteria | Pseudomonadales | Pseudomonadaceae | *Pseudomonas* | decontam package |
| ASV_680 | Proteobacteria | Gammaproteobacteria | Pseudomonadales | Pseudomonadaceae | *Pseudomonas* | decontam package |
| ASV_666 | Proteobacteria | Gammaproteobacteria | Pseudomonadales | Pseudomonadaceae | *Pseudomonas* | decontam package |
| ASV_198 | Proteobacteria | Gammaproteobacteria | Pseudomonadales | Pseudomonadaceae | *Pseudomonas* | decontam package |
| ASV_585 | Proteobacteria | Gammaproteobacteria | Pseudomonadales | Pseudomonadaceae | *Pseudomonas* | decontam package |
| ASV_2828 | Proteobacteria | Gammaproteobacteria | Xanthomonadales | Xanthomonadaceae | *Lysobacter* | decontam package |
| ASV_297 | Proteobacteria | Gammaproteobacteria | Xanthomonadales | Xanthomonadaceae | *Lysobacter* | decontam package |
| ASV_539 | Proteobacteria | Gammaproteobacteria | Xanthomonadales | Xanthomonadaceae | *Lysobacter* | decontam package |
| ASV_2241 | Proteobacteria | Gammaproteobacteria | Xanthomonadales | Xanthomonadaceae | *Lysobacter* | decontam package |
| ASV_230 | Proteobacteria | Gammaproteobacteria | Xanthomonadales | Xanthomonadaceae | *Lysobacter* | NTC profiles |
| ASV_224 | Proteobacteria | Gammaproteobacteria | Xanthomonadales | Xanthomonadaceae | *Stenotrophomonas* | decontam package |
| ASV_5984 | Spirochaetota | Spirochaetia | Spirochaetales | Spirochaetaceae | *Treponema* | decontam package |

*ASV. Amplicon sequence variant; NTC, No template control; A-N-P-R*: *Allorhizobium-Neorhizobium-Pararhizobium-Rhizobium*


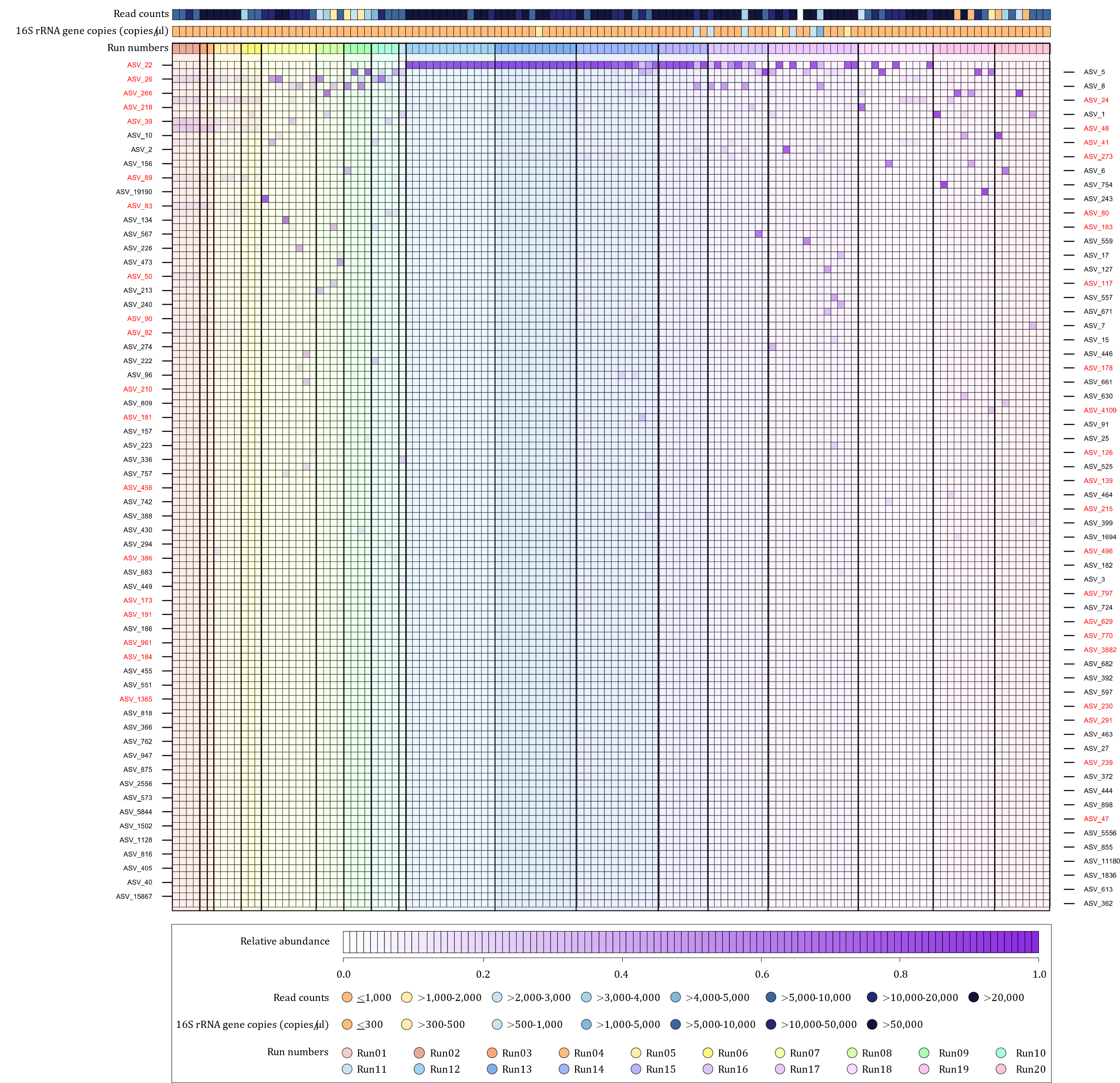


**Figure S11 Potential contaminant amplicon sequence variants (ASVs) identified from no template controls (NTCs)**

Heatmap of relative abundances of the top 120 ASVs present in NTCs (n=128), ordered according to run numbers. ASVs in red were manually selected as potential contaminants based on their relative abundances across the various runs.

**Table S7**. Compositional mean relative abundances of the top 15 amplicon sequence variants (ASVs) in each age category (0-3 months, >3-6 months, >6-12 months)

| 0-3 months (n=212) | | 4-6 months (n=232) | | 7-12 months (n=202) | |
| --- | --- | --- | --- | --- | --- |
| ASV | % | ASV | % | ASV | % |
| ASV1_*Moraxella* | 28.01 | ASV1_*Moraxella* | 40.97 | ASV1_ *Moraxella* | 46.68 |
| ASV2_ *Haemophilus* | 9.85 | ASV2_*Haemophilus* | 12.09 | ASV2_ *Haemophilus* | 14.76 |
| ASV5_Staphylococcus | 8.85 | ASV3_ *Haemophilus* | 9.66 | ASV3_ *Haemophilus* | 8.87 |
| ASV7_ *Corynebacterium* | 6.86 | ASV4_ *Corynebacterium* | 5.72 | ASV4_ *Corynebacterium* | 4.50 |
| ASV3_ *Haemophilus* | 6.50 | ASV8_*Streptococcus* | 4.66 | ASV6_*Dolosigranulum* | 3.86 |
| ASV4_*Corynebacterium* | 6.34 | ASV6_*Dolosigranulum* | 3.47 | ASV8_*Streptococcus* | 3.24 |
| ASV6_*Dolosigranulum* | 2.85 | ASV7_ *Corynebacterium* | 3.00 | ASV11_ *Moraxella* | 1.49 |
| ASV13_ *Neisseria* | 2.63 | ASV5_*Staphylococcus* | 1.94 | ASV5_*Staphylococcus* | 1.35 |
| ASV8_*Streptococcus* | 2.53 | ASV10_*Streptococcus* | 1.10 | ASV7_*Corynebacterium* | 1.14 |
| ASV10_*Streptococcus* | 2.03 | ASV11_ *Moraxella* | 1.02 | ASV18_*Neisseria* | 0.82 |
| ASV18_ *Neisseria* | 0.95 | ASV14_ *Haemophilus* | 0.95 | ASV28_ *Moraxella* | 0.66 |
| ASV61_*Chlamydia* | 0.90 | ASV21_ *Moraxella* | 0.84 | ASV21_ *Moraxella* | 0.64 |
| ASV42_*Prevotella* | 0.86 | ASV13_ *Neisseria* | 0.69 | ASV38_ *Moraxella* | 0.57 |
| ASV17_*Klebsiella* | 0.86 | ASV19_Family Neisseriaceae | 0.60 | ASV14_ *Haemophilus* | 0.57 |
| ASV25_*Streptococcus* | 0.82 | ASV71_ *Moraxella* | 0.46 | ASV20_ *Haemophilus* | 0.55 |
| other | 19.17 | other | 12.84 | other | 10.29 |

**Table S8**. Differential abundance testing for age at specimen collection.

| Taxon | MaAsLin2 coefficient | Number of NP specimens (%) | p-value | q-value^*^ | False discovery rate |
| --- | --- | --- | --- | --- | --- |
| ASV5_*Staphylococcus* | -1.70 | 228 (35) | 8.80E-25 | 8.10E-23 | 2.99E-23 |
| ASV7_*Corynebacterium* | -1.22 | 237 (37) | 8.80E-18 | 4.05E-16 | 1.50E-16 |
| ASV10_*Streptococcus* | -0.98 | 234 (36) | 8.35E-12 | 1.92E-10 | 7.09E-11 |
| ASV27_*Corynebacterium* | -0.40 | 73 (11) | 3.50E-05 | 4.02E-04 | 1.49E-04 |
| ASV40_*Gemella* | -0.55 | 180 (28) | 4.12E-05 | 4.21E-04 | 1.56E-04 |
| ASV34_*Corynebacterium* | -0.26 | 76 (12) | 9.63E-05 | 8.06E-04 | 2.98E-04 |
| ASV99_*Vitreoscilla* | -0.33 | 83 (13) | 8.67E-04 | 4.99E-03 | 1.84E-03 |
| ASV31_*Alloprevotella* | -0.34 | 126 (20) | 1.43E-03 | 7.75E-03 | 2.87E-03 |
| ASV25_*Streptococcus* | -0.34 | 132 (20) | 3.41E-03 | 1.74E-02 | 6.44E-03 |
| ASV13_*Neisseria* | -0.26 | 109 (17) | 1.10E-02 | 4.41E-02 | 1.63E-02 |
| ASV1_*Moraxella* | 1.39 | 569 (88) | 3.49E-16 | 1.07E-14 | 3.96E-15 |
| ASV6_*Dolosigranulum* | 1.08 | 419 (65) | 4.93E-10 | 9.07E-09 | 3.35E-09 |
| ASV3_*Haemophilus* | 1.13 | 239 (37) | 1.85E-08 | 2.84E-07 | 1.05E-07 |
| ASV2_*Haemophilus* | 0.62 | 390 (60) | 6.76E-04 | 4.36E-03 | 1.61E-03 |
| ASV8_*Streptococcus* | 0.47 | 315 (49) | 3.79E-03 | 1.75E-02 | 6.45E-03 |
| ASV4_*Corynebacterium* | 0.40 | 415 (64) | 2.46E-02 | 9.08E-02 | 3.35E-02 |
| ASV19_Family Neisseriaceae | 0.24 | 196 (30) | 7.51E-02 | 2.38E-01 | 8.80E-02 |

^*^Amplicon sequence variants (ASVs) with q-values <0.25 were deemed differentially abundant using Microbiome Multivariable Associations with Linear Models (MaAsLin2).

**Table S9.** Differential abundance testing for commencement of antibiotic therapy prior to specimen collection.

| Taxon | Antibiotic therapy prior to specimen collection | MaAsLin2 coefficient | Number of NP specimens (%) | p-value | q-value^*^ | False discovery rate |
| --- | --- | --- | --- | --- | --- | --- |
| ASV6_*Dolosigranulum* | Commenced >24 hours <7 days | -1.82 | 419 (65) | 5.0E-02 | 1.6E-01 | 6.1E-02 |
| ASV1_*Moraxella* | Commenced <24 hours | -1.45 | 569 (88) | 4.1E-02 | 1.4E-01 | 5.1E-02 |
| ASV186_*Lysobacter* | Commenced <24 hours | 0.55 | 77 (12) | 8.0E-02 | 2.4E-01 | 8.9E-02 |
| ASV31_*Alloprevotella* | Commenced <24 hours | 0.78 | 126 (20) | 8.9E-02 | 2.5E-01 | 9.1E-02 |
|  | Commenced >24 hours <7 days | 1.01 | 126 (20) | 8.1E-02 | 2.4E-01 | 8.9E-02 |
| ASV37_*Porphyromonas* | Commenced <24 hours | 1.07 | 115 (18) | 2.9E-02 | 1.0E-01 | 3.8E-02 |
| ASV99_*Vitreoscilla* | Commenced >24 hours <7 days | 1.37 | 83 (13) | 1.0E-02 | 4.3E-02 | 1.6E-02 |
| ASV19_Family Neisseriaceae | Commenced <24 hours | 1.66 | 196 (30) | 3.6E-03 | 1.7E-02 | 6.5E-03 |
|  | Commenced >24 hours <7 days | 2.71 | 196 (30) | 1.8E-04 | 1.4E-03 | 5.1E-04 |
| ASV40_*Gemella* | Commenced >24 hours <7 days | 1.90 | 180 (28) | 8.6E-03 | 3.8E-02 | 1.4E-02 |
| ASV10_*Streptococcus* | Commenced >24 hours <7 days | 3.04 | 234 (36) | 7.9E-05 | 7.3E-04 | 2.7E-04 |

^*^Amplicon sequence variants (ASVs) with q-values <0.25 were deemed differentially abundant using Microbiome Multivariable Associations with Linear Models (MaAsLin2).

**Table S10.** Bacterial taxa which were differentially abundant in children with more than one LRTI episode compared with those with only one LRTI episode.

| Taxon | Metadata variable | Regression coefficient^1^ | N (samples) | p-value^2^ | q-value |
| --- | --- | --- | --- | --- | --- |
| ASV2_*Haemophilus* | At least 2 LRTI episodes^3^ | 1.45 | 323 | 0.006 | 0.043 |
| ASV5_*Staphylococcus* | At least 2 LRTI episodes | 1.02 | 323 | 0.020 | 0.089 |
| ASV19_*Neisseriaceae* | At least 2 LRTI episodes | -1.13 | 323 | 0.001 | 0.008 |
| ASV10_*Streptococcus* | At least 2 LRTI episodes | -0.99 | 323 | 0.018 | 0.085 |

Logistic regression (MaAsLin2) model included age and antibiotic treatment as fixed effects.

*LRTI, Lower respiratory tract infection*

^1^Positive coefficient if positively associated with at least two LRTI episodes

^2^p-value <0.05 and q-value < 0.25 used to identify variables significantly associated with taxa; only significant results are shown

^3^Reference: samples from children with only one LRTI episode in the first year of life

**Table S11.** Five clusters based on the relative abundances of the top 25 ASVs in the dataset

|  | HAE_II | HAE__III | STA_COR | MOR | MIX |
| --- | --- | --- | --- | --- | --- |
|  | N=94 | N=56 | N=50 | N=278 | N=168 |
|  | n (%) | n (%) | n (%) | n (%) | n (%) |
| Age, median (IQR) | 153.5 (144) | 140 (100) | 62 (52) | 156 (135) | 99 (102) |
| LRTI cases |  |  |  |  |  |
| ambulatory | 45 (48) | 22 (39) | 14 (28) | 88 (32) | 68 (41) |
| hospitalized | 13 (14) | 8 (14) | 5 (10) | 28 (10) | 32 (19) |
| Viruses detected |  |  |  |  |  |
| RSV (A/B) | 11 (12) | 10 (18) | 5 (10) | 36 (13) | 22 (13) |
| Parainfluenza virus | 8 (9) | 2 (4) | 3 (6) | 17 (6) | 10 (6) |
| Enterovirus | 10 (11) | 7 (13) | 2 (4) | 17 (6) | 12 (7) |
| Influenza virus | 2 (2) | 1 (2) | 1 (2) | 10 (4) | 4 (2) |
| Adenovirus | 7 (7) | 4 (7) | 0 (0) | 32 (12) | 12 (7) |
| Human rhinovirus | 33 (35) | 22 (39) | 5 (10) | 83 (30) | 46 (27) |
| Cytomegalovirus | 49 (52) | 27 (48) | 11 (22) | 118 (43) | 58 (35) |

*LRTI, Lower respiratory tract infection; RSV, Respiratory syncytial virus*

**Table S12.** Associations between cluster membership and LRTI case status, controlling for age, bacterial load, and viral abundance

| Term | Estimate | Standard error | Statistic | p-value | Confidence (low) | Confidence (high) |
| --- | --- | --- | --- | --- | --- | --- |
| (Intercept) | -1.60216 | 0.535038 | -2.99447 | 0.002749 | -2.66217 | -0.56175 |
| HAE_III | -0.05976 | 0.288539 | -0.20712 | 0.835912 | -0.62592 | 0.507194 |
| STA_COR | -0.71598 | 0.21927 | -3.26529 | 0.001094 | -1.14913 | -0.28863 |
| MOR | -0.65314 | 0.39356 | -1.65956 | 0.097003 | -1.44635 | 0.106695 |
| MIX | -0.298 | 0.332236 | -0.89696 | 0.36974 | -0.95105 | 0.355075 |
| RSV (A/B) | 1.61E-08 | 6.66E-09 | 2.425156 | 0.015302 | 5.72E-09 | 3.21E-08 |
| Parainfluenza virus | 8.91E-08 | 6.81E-08 | 1.30845 | 0.190721 | 8.81E-09 | 2.7E-07 |
| Enterovirus virus | 5.03E-10 | 4.13E-09 | 0.121873 | 0.903 | -8.5E-09 | 1E-08 |
| Adenovirus | 2.53E-10 | 2.8E-10 | 0.902509 | 0.366787 | -2.6E-10 | 1.03E-09 |
| Human rhinovirus | 1.2E-09 | 4.58E-09 | 0.261825 | 0.793456 | -8E-09 | 1.15E-08 |
| Cytomegalovirus | 2.48E-07 | 1.28E-07 | 1.939885 | 0.052394 | 5.93E-08 | 5.74E-07 |
| Age | 0.000226 | 0.000939 | 0.240686 | 0.809799 | -0.00162 | 0.002072 |
| Bacterial load | 0.44036 | 0.132022 | 3.33551 | 0.000851 | 0.183949 | 0.702255 |

**Supplementary references**

1. Zar HJ, Barnett W, Myer L, Stein DJ, Nicol MP. Investigating the early-life determinants of illness in Africa: the Drakenstein Child Health Study. Thorax **2014**; 0:1–3. Available at: http://www.ncbi.nlm.nih.gov/pubmed/25228292. Accessed 21 September 2014.

2. Fenton TR, Nasser R, Eliasziw M, Kim JH, Bilan D, Sauve R. Validating the weight gain of preterm infants between the reference growth curve of the fetus and the term infant. BMC Pediatr **2013**; 13.

3. Fenton TR, Kim JH. A systematic review and meta-analysis to revise the Fenton growth chart for preterm infants. BMC Pediatr **2013**; 13.

4. Gray DiM, Wedderburn CJ, MacGinty RP, et al. Impact of HIV and antiretroviral drug exposure on lung growth and function over 2 years in an African Birth Cohort. Aids **2020**; 34:549–558.

5. Zar HJ, Pellowski JA, Cohen S, et al. Maternal health and birth outcomes in a South African birth cohort study. PLoS One **2019**; 14:1–16.

6. Claassen-Weitz S, Gardner-Lubbe S, Mwaikono KS, du Toit E, Zar HJ, Nicol MP. Optimizing 16S rRNA gene profile analysis from low biomass nasopharyngeal and induced sputum specimens. BMC Microbiol **2020**; 20:113.

7. Bogaert D, Keijser B, Huse S, et al. Variability and diversity of nasopharyngeal microbiota in children: A metagenomic analysis. PLoS One **2011**; 6.

8. Claassen-Weitz S, Gardner-Lubbe S, Nicol P, et al. HIV-exposure, early life feeding practices and delivery mode impacts on faecal bacterial profiles in a South African birth cohort. Sci Rep **2018**; 8:1–15. Available at: http://dx.doi.org/10.1038/s41598-018-22244-6.

9. Caporaso JG, Lauber CL, Walters WA, et al. Global patterns of 16S rRNA diversity at a depth of millions of sequences per sample. PNAS **2010**; 108:4516–4522.

10. Illumina Proprietary. MiSeq ® Reagent Kit v3 Reagent Preparation Guide. 2013; :1–14. Available at: http://supportres.illumina.com/documents/documentation/system_documentation/miseq/miseq-reagent-kit-v3-reagent-prep-guide-15044983-b.pdf.

11. Illumina Proprietary. MiSeq ® System User Guide. 2014; :1–94. Available at: http://supportres.illumina.com/documents/documentation/system_documentation/miseq/miseq-system-user-guide-15027617-n.pdf.

12. Andrews S. FastQC: a quality control tool for high throughput sequence data. 2010; Available at: http://www.bioinformatics.babraham.ac.uk/projects/fastqc.

13. Ewels P, Magnusson M, Lundin S, Käller M. MultiQC: Summarize analysis results for multiple tools and samples in a single report. Bioinformatics **2016**; 32:3047–3048.

14. Callahan BJ, McMurdie PJ, Rosen MJ, Han AW, Johnson, Amy Jo A Holmes SP. DADA2: High resolution sample inference from Illumina amplicon data. Nat Methods **2016**; 13:581–3.

15. Tommaso P Di, Chatzou M, Floden EW, Barja PP, Palumbo E, Notredame C. Nextflow enables reproducible computational workflows. Nat Biotechnol **2017**; 35:316–319.

16. Cole JR, Wang Q, Fish JA, et al. Ribosomal Database Project: Data and tools for high throughput rRNA analysis. Nucleic Acids Res **2014**; 42:633–642.

17. Callahan BJ. RDP taxonomic training data formatted for DADA2 (RDP trainset 16/release 11.5). 2017. Available at: https://zenodo.org/record/801828#.X7VWKs7itdg.

18. Quast C, Pruesse E, Yilmaz P, et al. The SILVA ribosomal RNA gene database project: improved data processing and web-based tools. Nucleic Acids Res **2013**; 41:D590-6. Available at: http://www.pubmedcentral.nih.gov/articlerender.fcgi?artid=3531112&tool=pmcentrez&rendertype=abstract. Accessed 10 July 2014.

19. R Core Team. R Foundation for Statistical Computing. R: A language and environment for statistical computing. 2018; Available at: https://www.r-project.org/.

20. RStudio Team. RStudio: Integrated development environment for R. 2021; Available at: http://www.rstudio.org/.

21. Davis NM, Proctor D, Holmes SP, Relman DA, Callahan BJ. Simple statistical identification and removal of contaminant sequences in marker-gene and metagenomics data. bioRxiv **2017**; :221499. Available at: https://www.biorxiv.org/content/early/2018/07/25/221499.
